# Supplementary material for: Functional regulation of aquaporin dynamics by lipid bilayer composition
Source: Nat Commun. 2024 Feb 28;15:1848. doi: 10.1038/s41467-024-46027-y (PMC10901782; doi:10.1038/s41467-024-46027-y)
Supplement: Supplementary file 1 — Supplementary Information [file 41467_2024_46027_MOESM1_ESM.pdf]

# Supplementary Information

# Functional regulation of aquaporin dynamics by lipid bilayer composition

*Anh T. P. Nguyen,*[1] *Austin T. Weigle,*[2] *and Diwakar Shukla**[,1,3,4,5]

[1]Department of Chemical and Biomolecular Engineering, [2]Department of Chemistry, [3]Center for Biophysics and Computational Biology, [4]Department of Bioengineering, [5]Department of Plant Biology, University of Illinois at Urbana-Champaign, Urbana, IL 61801, United States

*Email: diwakar@illinois.edu

# Table of Contents


| | | |
|---|---|---|
| **S. Table 1** | Final Markov state model (MSM) parameters and total simulation time for each SoPIP2;1 lipid bilayer system | S-5 |
| **S. Table 2** | SoPIP2:POPC adaptive sampling summary | S-6 |
| **S. Table 3** | SoPIP2:POPE adaptive sampling summary | S-7 |
| **S. Table 4** | SoPIP2:POPG adaptive sampling summary | S-8 |
| **S. Table 5** | SoPIP2:PLPC adaptive sampling summary | S-9 |
| **S. Table 6** | SoPIP2:PLPE adaptive sampling summary | S-10 |
| **S. Table 7** | SoPIP2:PLPG adaptive sampling summary | S-11 |
| **S. Table 8** | SoPIP2:LLPC adaptive sampling summary | S-12 |
| **S. Table 9** | SoPIP2:LLPE adaptive sampling summary | S-13 |
| **S. Table 10** | SoPIP2:LLPG adaptive sampling summary | S-14 |
| **S. Table 11** | SoPIP2:complex adaptive sampling summary | S-15 |
| **S. Figure 1** | SoPIP2;1 topology | S-16 |
| **S. Figure 2** | Distance features and their correlation to tICA components generated by RRCS and spectral oASIS workflow | S-17 |
| **S. Figure 3** | Implied timescale plots for the SoPIP2:bilayer MSMs capturing the transitions for loop D conformational change. | S-18 |
| **S. Figure 4** | Raw counts versus MSM population for each microstate cluster of SoPIP2:bilayer conformational dynamics | S-19 |
| **S. Figure 5** | Chapman–Kolmogorov test for the MSM of SoPIP2:POPC conformational dynamics | S-20 |
| **S. Figure 6** | Chapman–Kolmogorov test for the MSM of SoPIP2:POPE conformational dynamics | S-21 |
| **S. Figure 7** | Chapman–Kolmogorov test for the MSM of SoPIP2:POPG conformational dynamics | S-22 |

**S. Figure 8** Chapman–Kolmogorov test for the MSM of SoPIP2:PLPC conformational dynamics S-23

**S. Figure 9** Chapman–Kolmogorov test for the MSM of SoPIP2:PLPE conformational dynamics S-24

**S. Figure 10** Chapman–Kolmogorov test for the MSM of SoPIP2:PLPG conformational dynamics S-25

**S. Figure 11** Chapman–Kolmogorov test for the MSM of SoPIP2:LLPC conformational dynamics S-26

**S. Figure 12** Chapman–Kolmogorov test for the MSM of SoPIP2:LLPE conformational dynamics S-27

**S. Figure 13** Chapman–Kolmogorov test for the MSM of SoPIP2:LLPG conformational dynamics S-28

**S. Figure 14** Chapman–Kolmogorov test for the MSM of SoPIP2:complex conformational dynamics S-29

**S. Figure 15** Free energy error from the 200-sample bootstrapping protocol for each SoPIP2:bilayer system S-30

**S. Figure 16** Data distributions for MFPT calculations of open-to-closed and closed-to-open transitions S-31

**S. Figure 17** Selected continuous trajectories for the water transport analysis based on tICA macrostate identification S-32

**S. Figure 18** Area per lipid (APL) cumulative average from initial 1 μs production run S-33

**S. Figure 19** Relationship between number of waters transported and the rate of transport S-34

**S. Figure 20** Pore cavity structure with respect to loop D conformation throughout different types of example transport cases S-35

**S. Figure 21** Residence time of water molecules at each slice of the SoPIP2;1 pore S-36

**S. Figure 22** Comparison of average SoPIP2;1 lipid contact probability along the protein-lipid interface against MemProtMD simulated aquaporins (Part 1) S-37

**S. Figure 23** Comparison of average SoPIP2;1 lipid contact probability along the protein-lipid interface against MemProtMD simulated aquaporins (Part 2) S-38

**S. Figure 24** Comparison of average SoPIP2;1 lipid contact probability along the protein-lipid interface against MemProtMD simulated aquaporins (Part 3) S-39

**S. Figure 25** PyLipID average residence time for SoPIP2:lipid interactions along the open state S-40

**S. Figure 26** PyLipID average residence time for SoPIP2:lipid interactions along intermediate states S-41

**S. Figure 27** PyLipID average residence time for SoPIP2:lipid interactions along the closed state S-42

**S. Figure 28** Radial fingerprints for loop D lipid binding interactions S-43

**S. Figure 29** Dissecting influence of lipid headgroups or acyl chains on SoPIP2;1 protein function S-44

**S. Figure 30** Number of waters imported versus average lipid order parameter for all selected trajectories belonging to each SoPIP2:bilayer macrostate S-45

**S. Figure 31** Number of waters imported versus the average lipid order parameter of the annular shell lipids of the open states S-46

**S. Figure 32** Correlation between the thickness and order parameter of the lipids for each homogeneous SoPIP2:bilayer macrostate S-47

**S. Figure 33** Number of waters imported versus thickness for each SoPIP2:bilayer macrostate S-48

**Reporting Statistics** S-49

**Supplementary Table 1. Final Markov state model (MSM) parameters and total simulation time for each SoPIP2;1 lipid bilayer system**

| Bilayer | Features | tIC dimensions | Clusters | MSM lag time (ns) | Total simulation time ($\mu$s) |
|---|---|---|---|---|---|
| POPC | 40 | 5 | 400 | 4 | 19.23 |
| POPE | 25 | 12 | 300 | 4 | 31.59 |
| POPG | 30 | 7 | 500 | 4 | 20.00 |
| PLPC | 30 | 7 | 700 | 6 | 54.71 |
| PLPE | 25 | 8 | 500 | 6 | 29.50 |
| PLPG | 40 | 4 | 500 | 4 | 26.30 |
| LLPC | 40 | 4 | 300 | 4 | 19.77 |
| LLPE | 25 | 7 | 400 | 6 | 49.50 |
| LLPG | 40 | 4 | 600 | 8 | 23.47 |
| Complex | 20 | 6 | 400 | 6 | 41.62 |

**Supplementary Table 2. SoPIP2:POPC adaptive sampling summary.** The first round of simulation, “Round 1”, consists of a single 1 $\mu$s trajectory originated from each of the crystal structure embeddings. This single trajectory took a few “rounds” to complete due to wallclock time limitations. The first “true” round of adaptive sampling is labelled as “Round 4”. Each trajectory within a round of sampling outside of “Round 1” is set to be 100 ns in length.

| POPC Round | Parallel Simulations | | Trajectory Length (ns) | | Round Simulation Time (ns) | | Aggregate Time (μs) |
|---|---|---|---|---|---|---|---|
| | 2b5f (open) | 1z98 (closed) | 2b5f (open) | 1z98 (closed) | 2b5f (open) | 1z98 (closed) | |
| ***1*** | 1 | 1 | 1000 | 1000 | 1028.95 | 1200.00 | 2.23 |
| ***4*** | 2 | 8 | 100 | 100 | 200.00 | 800.00 | 1.00 |
| ***5*** | 3 | 7 | 100 | 100 | 300.00 | 700.00 | 1.00 |
| ***6*** | 10 | 10 | 100 | 100 | 1000.00 | 1000.00 | 2.00 |
| ***7*** | 10 | 10 | 100 | 100 | 1000.00 | 1000.00 | 2.00 |
| ***8*** | 10 | 10 | 100 | 100 | 1000.00 | 1000.00 | 2.00 |
| ***9*** | 5 | 5 | 100 | 100 | 500.00 | 500.00 | 1.00 |
| ***10*** | 5 | 5 | 100 | 100 | 500.00 | 500.00 | 1.00 |
| ***11*** | 10 | 10 | 100 | 100 | 1000.00 | 1000.00 | 2.00 |
| ***12*** | 0 | 10 | 100 | 100 | 0.00 | 1000.00 | 1.00 |
| ***13*** | 10 | 10 | 100 | 100 | 1000.00 | 1000.00 | 2.00 |
| ***14*** | 10 | 10 | 100 | 100 | 1000.00 | 1000.00 | 2.00 |
| **Total Simulation Time:** *19.23 μs* | | | | | | | |

**Supplementary Table 3. SoPIP2:POPE adaptive sampling summary.** The first round of simulation, "Round 1", consists of a single 1 $\mu$s trajectory originated from each of the crystal structure embeddings. This single trajectory took a few "rounds" to complete due to wallclock time limitations. The first "true" round of adaptive sampling is labelled as "Round 4". Each trajectory within a round of sampling outside of "Round 1" is set to be 100 ns in length.

| POPE Round | Parallel Simulations | | Trajectory Length (ns) | | Round Simulation Time (ns) | | Aggregate Time (μs) |
|---|---|---|---|---|---|---|---|
| | 2b5f (open) | 1z98 (closed) | 2b5f (open) | 1z98 (closed) | 2b5f (open) | 1z98 (closed) | |
| ***1*** | 1 | 1 | 1000 | 1000 | 1000.00 | 1000.00 | 2.00 |
| ***4*** | 8 | 2 | 100 | 100 | 800.00 | 169.25 | 0.97 |
| ***5*** | 8 | 3 | 100 | 100 | 700.00 | 301.06 | 1.00 |
| ***6*** | 10 | 10 | 100 | 100 | 1000.00 | 1000.00 | 2.00 |
| ***7*** | 10 | 10 | 100 | 100 | 1000.00 | 921.26 | 1.92 |
| ***8*** | 10 | 10 | 100 | 100 | 1000.00 | 1000.00 | 2.00 |
| ***9*** | 10 | 10 | 100 | 100 | 1000.00 | 1000.00 | 2.00 |
| ***10*** | 10 | 10 | 100 | 100 | 1000.00 | 1000.00 | 2.00 |
| ***11*** | 10 | 10 | 100 | 100 | 1000.00 | 1000.00 | 2.00 |
| ***12*** | 10 | 10 | 100 | 100 | 1000.00 | 1000.00 | 2.00 |
| ***13*** | 7 | 10 | 100 | 100 | 700.00 | 1000.00 | 1.70 |
| ***14*** | 10 | 10 | 100 | 100 | 1000.00 | 1000.00 | 2.00 |
| ***15*** | 10 | 10 | 100 | 100 | 1000.00 | 1000.00 | 2.00 |
| ***16*** | 10 | 10 | 100 | 100 | 1000.00 | 1000.00 | 2.00 |
| ***17*** | 0 | 10 | 100 | 100 | 0.00 | 1000.00 | 1.00 |
| ***18*** | 0 | 10 | 100 | 100 | 0.00 | 1000.00 | 1.00 |
| ***19*** | 10 | 10 | 100 | 100 | 1000.00 | 1000.00 | 2.00 |
| ***20*** | 10 | 0 | 100 | 100 | 1000.00 | 0.00 | 1.00 |
| ***21*** | 10 | 0 | 100 | 100 | 1000.00 | 0.00 | 1.00 |
| **Total Simulation Time:** *31.59 μs* | | | | | | | |

**Supplementary Table 4. SoPIP2:POPG adaptive sampling summary.** The first round of simulation, “Round 1”, consists of a single 1 $\mu$s trajectory originated from each of the crystal structure embeddings. This single trajectory took a few “rounds” to complete due to wallclock time limitations. The first “true” round of adaptive sampling is labelled as “Round 4”. Each trajectory within a round of sampling outside of “Round 1” is set to be 100 ns in length.

| POPG Round | Parallel Simulations | | Trajectory Length (ns) | | Round Simulation Time (ns) | | Aggregate Time (μs) |
|---|---|---|---|---|---|---|---|
| | 2b5f (open) | 1z98 (closed) | 2b5f (open) | 1z98 (closed) | 2b5f (open) | 1z98 (closed) | |
| ***1*** | 1 | 1 | 1000 | 1000 | 1000.00 | 1000.00 | 2.00 |
| ***4*** | 5 | 5 | 100 | 100 | 500.00 | 500.00 | 1.00 |
| ***5*** | 9 | 1 | 100 | 100 | 900.00 | 100.00 | 1.00 |
| ***6*** | 7 | 3 | 100 | 100 | 700.00 | 300.00 | 1.00 |
| ***7*** | 5 | 5 | 100 | 100 | 500.00 | 500.00 | 1.00 |
| ***8*** | 10 | 10 | 100 | 100 | 1000.00 | 1000.00 | 2.00 |
| ***9*** | 10 | 10 | 100 | 100 | 1000.00 | 1000.00 | 2.00 |
| ***10*** | 20 | 20 | 100 | 100 | 2000.00 | 2000.00 | 4.00 |
| ***11*** | 20 | 20 | 100 | 100 | 2000.00 | 2000.00 | 4.00 |
| ***12*** | 10 | 10 | 100 | 100 | 1000.00 | 1000.00 | 2.00 |
| **Total Simulation Time:** *20.00 μs* | | | | | | | |

**Supplementary Table 5. SoPIP2:PLPC adaptive sampling summary.** The first round of simulation, "Round 1", consists of a single 1 $\mu$s trajectory originated from each of the crystal structure embeddings. This single trajectory took a few "rounds" to complete due to wallclock time limitations. The first "true" round of adaptive sampling is labelled as "Round 4". Each trajectory within a round of sampling outside of "Round 1" is set to be 100 ns in length.

| PLPC | Parallel Simulations | | Trajectory Length (ns) | | Round Simulation Time (ns) | | Aggregate Time (μs) |
|---|---|---|---|---|---|---|---|
| Round | 2b5f (open) | 1z98 (closed) | 2b5f (open) | 1z98 (closed) | 2b5f (open) | 1z98 (closed) | |
| ***1*** | 1 | 1 | 1000 | 1000 | 1000.00 | 1000.00 | 2.00 |
| ***4*** | 2 | 8 | 100 | 100 | 200.00 | 800.00 | 1.00 |
| ***5*** | 10 | 10 | 100 | 100 | 1000.00 | 1000.00 | 2.00 |
| ***6*** | 10 | 10 | 100 | 100 | 1000.00 | 1000.00 | 2.00 |
| ***7*** | 10 | 10 | 100 | 100 | 1000.00 | 1000.00 | 2.00 |
| ***8*** | 5 | 5 | 100 | 100 | 500.00 | 500.00 | 1.00 |
| ***9*** | 10 | 10 | 100 | 100 | 1000.00 | 1000.00 | 2.00 |
| ***10*** | 10 | 10 | 100 | 100 | 1000.00 | 1000.00 | 2.00 |
| ***11*** | 10 | 10 | 100 | 100 | 1000.00 | 1000.00 | 2.00 |
| ***12*** | 10 | 10 | 100 | 100 | 1000.00 | 1000.00 | 2.00 |
| ***13*** | 10 | 10 | 100 | 100 | 1000.00 | 1000.00 | 2.00 |
| ***14*** | 10 | 10 | 100 | 100 | 1000.00 | 1000.00 | 2.00 |
| ***15*** | 10 | 10 | 100 | 100 | 1000.00 | 1000.00 | 2.00 |
| ***16*** | 10 | 10 | 100 | 100 | 1000.00 | 1000.00 | 2.00 |
| ***17*** | 10 | 10 | 100 | 100 | 1000.00 | 1000.00 | 2.00 |
| ***18*** | 10 | 10 | 100 | 100 | 1000.00 | 1000.00 | 2.00 |
| ***19*** | 10 | 10 | 100 | 100 | 1000.00 | 1000.00 | 2.00 |
| ***20*** | 10 | 10 | 100 | 100 | 863.68 | 1000.00 | 1.86 |
| ***21*** | 10 | 10 | 100 | 100 | 996.34 | 952.20 | 1.95 |
| ***22*** | 10 | 10 | 100 | 100 | 1000.00 | 1000.00 | 2.00 |
| ***23*** | 10 | 10 | 100 | 100 | 1000.00 | 1000.00 | 2.00 |
| ***24*** | 10 | 10 | 100 | 100 | 1000.00 | 1000.00 | 2.00 |
| ***25*** | 10 | 0 | 100 | 100 | 1000.00 | 0.00 | 1.00 |
| ***26*** | 10 | 0 | 100 | 100 | 1000.00 | 0.00 | 1.00 |
| ***27*** | 10 | 0 | 100 | 100 | 1000.00 | 0.00 | 1.00 |
| ***28*** | 10 | 10 | 100 | 100 | 1000.00 | 1000.00 | 2.00 |
| ***29*** | 15 | 15 | 100 | 100 | 1500.00 | 1500.00 | 3.00 |
| ***30*** | 9 | 10 | 100 | 100 | 900.00 | 1000.00 | 1.90 |
| ***31*** | 15 | 15 | 100 | 100 | 1500.00 | 1500.00 | 3.00 |
| **Total Simulation Time:** *54.71 μs* | | | | | | | |

**Supplementary Table 6. SoPIP2:PLPE adaptive sampling summary.** The first round of simulation, "Round 1", consists of a single 1 $\mu$s trajectory originated from each of the crystal structure embeddings. This single trajectory took a few "rounds" to complete due to wallclock time limitations. The first "true" round of adaptive sampling is labelled as "Round 4". Each trajectory within a round of sampling outside of "Round 1" is set to be 100 ns in length.

| PLPE Round | Parallel Simulations | | Trajectory Length (ns) | | Round Simulation Time (ns) | | Aggregate Time (μs) |
|---|---|---|---|---|---|---|---|
| | 2b5f (open) | 1z98 (closed) | 2b5f (open) | 1z98 (closed) | 2b5f (open) | 1z98 (closed) | |
| ***1*** | 1 | 1 | 1000 | 1000 | 1000.00 | 1000.00 | 2.00 |
| ***4*** | 10 | 10 | 100 | 100 | 1000.00 | 1000.00 | 2.00 |
| ***5*** | 10 | 0 | 100 | 100 | 1000.00 | 0.00 | 1.00 |
| ***6*** | 10 | 10 | 100 | 100 | 1000.00 | 1000.00 | 2.00 |
| ***7*** | 10 | 10 | 100 | 100 | 1000.00 | 1000.00 | 2.00 |
| ***8*** | 10 | 10 | 100 | 100 | 1000.00 | 1000.00 | 2.00 |
| ***9*** | 10 | 10 | 100 | 100 | 1000.00 | 1000.00 | 2.00 |
| ***10*** | 5 | 5 | 100 | 100 | 500.00 | 500.00 | 1.00 |
| ***11*** | 10 | 10 | 100 | 100 | 1000.00 | 1000.00 | 2.00 |
| ***12*** | 10 | 10 | 100 | 100 | 1000.00 | 1000.00 | 2.00 |
| ***13*** | 10 | 10 | 100 | 100 | 1000.00 | 1000.00 | 2.00 |
| ***14*** | 10 | 10 | 100 | 100 | 1000.00 | 1000.00 | 2.00 |
| ***15*** | 10 | 10 | 100 | 100 | 1000.00 | 1000.00 | 2.00 |
| ***16*** | 10 | 10 | 100 | 100 | 1000.00 | 1000.00 | 2.00 |
| ***17*** | 15 | 0 | 100 | 100 | 1500.00 | 0.00 | 1.50 |
| ***18*** | 20 | 0 | 100 | 100 | 2000.00 | 0.00 | 2.00 |
| **Total Simulation Time:** *29.50 μs* | | | | | | | |

**Supplementary Table 7. SoPIP2:PLPG adaptive sampling summary.** The first round of simulation, “Round 1”, consists of a single 1 $\mu$s trajectory originated from each of the crystal structure embeddings. This single trajectory took a few “rounds” to complete due to wallclock time limitations. The first “true” round of adaptive sampling is labelled as “Round 4”. Each trajectory within a round of sampling outside of “Round 1” is set to be 100 ns in length.

| **PLPG** | **Parallel Simulations** | | **Trajectory Length (ns)** | | **Round Simulation Time (ns)** | | **Aggregate Time (μs)** |
|---|---|---|---|---|---|---|---|
| **Round** | **2b5f (open)** | **1z98 (closed)** | **2b5f (open)** | **1z98 (closed)** | **2b5f (open)** | **1z98 (closed)** | |
| ***1*** | 1 | 1 | 1000 | 1000 | 1000.00 | 900.00 | 1.90 |
| ***4*** | 8 | 2 | 100 | 100 | 800.00 | 200.00 | 1.00 |
| ***5*** | 10 | 10 | 100 | 100 | 1000.00 | 1000.00 | 2.00 |
| ***6*** | 10 | 0 | 100 | 100 | 1000.00 | 0.00 | 1.00 |
| ***7*** | 10 | 0 | 100 | 100 | 1000.00 | 0.00 | 1.00 |
| ***8*** | 10 | 0 | 100 | 100 | 1000.00 | 0.00 | 1.00 |
| ***9*** | 10 | 0 | 100 | 100 | 1000.00 | 0.00 | 1.00 |
| ***10*** | 7 | 3 | 100 | 100 | 700.00 | 300.00 | 1.00 |
| ***11*** | 20 | 0 | 100 | 100 | 2000.00 | 0.00 | 2.00 |
| ***12*** | 20 | 0 | 100 | 100 | 2000.00 | 0.00 | 2.00 |
| ***13*** | 10 | 0 | 100 | 100 | 1000.00 | 0.00 | 1.00 |
| ***14*** | 10 | 0 | 100 | 100 | 1000.00 | 0.00 | 1.00 |
| ***15*** | 0 | 20 | 100 | 100 | 0.00 | 2000.00 | 2.00 |
| ***16*** | 15 | 15 | 100 | 100 | 1500.00 | 1500.00 | 3.00 |
| ***17*** | 10 | 20 | 100 | 100 | 1000.00 | 2000.00 | 3.00 |
| ***18*** | 10 | 15 | 100 | 100 | 903.28 | 1500.00 | 2.40 |
| **Total Simulation Time:** *26.30 μs* | | | | | | | |

**Supplementary Table 8. SoPIP2:LLPC adaptive sampling summary.** The first round of simulation, "Round 1", consists of a single 1 $\mu$s trajectory originated from each of the crystal structure embeddings. This single trajectory took a few "rounds" to complete due to wallclock time limitations. The first "true" round of adaptive sampling is labelled as "Round 4". Each trajectory within a round of sampling outside of "Round 1" is set to be 100 ns in length.

| LLPC Round | Parallel Simulations | | Trajectory Length (ns) | | Round Simulation Time (ns) | | Aggregate Time (μs) |
|---|---|---|---|---|---|---|---|
| | 2b5f (open) | 1z98 (closed) | 2b5f (open) | 1z98 (closed) | 2b5f (open) | 1z98 (closed) | |
| ***1*** | 1 | 1 | 1000 | 1000 | 914.33 | 1000.00 | 1.91 |
| ***4*** | 7 | 3 | 100 | 100 | 700.00 | 300.00 | 1.00 |
| ***5*** | 2 | 8 | 100 | 100 | 200.00 | 798.50 | 1.00 |
| ***6*** | 10 | 10 | 100 | 100 | 716.30 | 936.63 | 1.65 |
| ***7*** | 10 | 5 | 100 | 100 | 940.12 | 438.75 | 1.38 |
| ***8*** | 10 | 10 | 100 | 100 | 842.77 | 986.74 | 1.83 |
| ***9*** | 10 | 10 | 100 | 100 | 1000.00 | 1000.00 | 2.00 |
| ***10*** | 10 | 10 | 100 | 100 | 1000.00 | 1000.00 | 2.00 |
| ***11*** | 10 | 10 | 100 | 100 | 1000.00 | 1000.00 | 2.00 |
| ***12*** | 10 | 0 | 100 | 100 | 1000.00 | 0.00 | 1.00 |
| ***13*** | 10 | 10 | 100 | 100 | 1000.00 | 1000.00 | 2.00 |
| ***14*** | 10 | 10 | 100 | 100 | 1000.00 | 1000.00 | 2.00 |
| **Total Simulation Time:** *19.77 μs* | | | | | | | |

**Supplementary Table 9. SoPIP2:LLPE adaptive sampling summary.** The first round of simulation, "Round 1", consists of a single 1 $\mu$s trajectory originated from each of the crystal structure embeddings. This single trajectory took a few "rounds" to complete due to wallclock time limitations. The first "true" round of adaptive sampling is labelled as "Round 4". Each trajectory within a round of sampling outside of "Round 1" is set to be 100 ns in length.

| LLPE Round | Parallel Simulations | | Trajectory Length (ns) | | Round Simulation Time (ns) | | Aggregate Time (μs) |
|---|---|---|---|---|---|---|---|
| | 2b5f (open) | 1z98 (closed) | 2b5f (open) | 1z98 (closed) | 2b5f (open) | 1z98 (closed) | |
| ***1*** | 1 | 1 | 1000 | 1000 | 1736.20 | 1000.00 | 2.74 |
| ***4*** | 10 | 10 | 100 | 100 | 1000.00 | 1000.00 | 2.00 |
| ***5*** | 10 | 10 | 100 | 100 | 1000.00 | 1000.00 | 2.00 |
| ***6*** | 10 | 10 | 100 | 100 | 1000.00 | 1000.00 | 2.00 |
| ***7*** | 10 | 10 | 100 | 100 | 1000.00 | 1000.00 | 2.00 |
| ***8*** | 4 | 5 | 100 | 100 | 400.00 | 500.00 | 0.90 |
| ***9*** | 8 | 8 | 100 | 100 | 800.00 | 800.00 | 1.60 |
| ***10*** | 13 | 5 | 100 | 100 | 1300.00 | 500.00 | 1.80 |
| ***11*** | 10 | 10 | 100 | 100 | 1000.00 | 1000.00 | 2.00 |
| ***12*** | 10 | 10 | 100 | 100 | 1000.00 | 1000.00 | 2.00 |
| ***13*** | 10 | 10 | 100 | 100 | 1000.00 | 1000.00 | 2.00 |
| ***14*** | 10 | 10 | 100 | 100 | 1000.00 | 1000.00 | 2.00 |
| ***15*** | 10 | 10 | 100 | 100 | 1000.00 | 1000.00 | 2.00 |
| ***16*** | 10 | 10 | 100 | 100 | 1000.00 | 1000.00 | 2.00 |
| ***17*** | 10 | 10 | 100 | 100 | 1000.00 | 1000.00 | 2.00 |
| ***18*** | 10 | 10 | 100 | 100 | 1000.00 | 1000.00 | 2.00 |
| ***19*** | 10 | 10 | 100 | 100 | 1000.00 | 1000.00 | 2.00 |
| ***20*** | 10 | 10 | 100 | 100 | 1000.00 | 1000.00 | 2.00 |
| ***21*** | 10 | 10 | 100 | 100 | 1000.00 | 1000.00 | 2.00 |
| ***22*** | 10 | 10 | 100 | 100 | 1000.00 | 1000.00 | 2.00 |
| ***23*** | 10 | 10 | 100 | 100 | 1000.00 | 963.42 | 1.96 |
| ***24*** | 10 | 10 | 100 | 100 | 1000.00 | 1000.00 | 2.00 |
| ***25*** | 10 | 10 | 100 | 100 | 1000.00 | 1000.00 | 2.00 |
| ***26*** | 0 | 10 | 100 | 100 | 0.00 | 1000.00 | 1.00 |
| ***27*** | 0 | 20 | 100 | 100 | 0.00 | 2000.00 | 2.00 |
| ***28*** | 15 | 0 | 100 | 100 | 1500.00 | 0.00 | 1.50 |
| **Total Simulation Time:** *49.50 μs* | | | | | | | |

**Supplementary Table 10. SoPIP2:LLPG adaptive sampling summary.** The first round of simulation, "Round 1", consists of a single 1 $\mu$s trajectory originated from each of the crystal structure embeddings. This single trajectory took a few "rounds" to complete due to wallclock time limitations. The first "true" round of adaptive sampling is labelled as "Round 4". Each trajectory within a round of sampling outside of "Round 1" is set to be 100 ns in length.

| LLPG Round | Parallel Simulations | | Trajectory Length (ns) | | Round Simulation Time (ns) | | Aggregate Time (μs) |
|---|---|---|---|---|---|---|---|
| | 2b5f (open) | 1z98 (closed) | 2b5f (open) | 1z98 (closed) | 2b5f (open) | 1z98 (closed) | |
| ***1*** | 1 | 1 | 1000 | 1000 | 1000.00 | 1000.00 | 2.00 |
| ***4*** | 10 | 10 | 100 | 100 | 1000.00 | 1000.00 | 2.00 |
| ***5*** | 10 | 0 | 100 | 100 | 1000.00 | 0.00 | 1.00 |
| ***6*** | 10 | 10 | 100 | 100 | 1000.00 | 1000.00 | 2.00 |
| ***7*** | 10 | 10 | 100 | 100 | 1000.00 | 1000.00 | 2.00 |
| ***8*** | 10 | 10 | 100 | 100 | 1000.00 | 1000.00 | 2.00 |
| ***9*** | 10 | 10 | 100 | 100 | 1000.00 | 1000.00 | 2.00 |
| ***10*** | 5 | 5 | 100 | 100 | 500.00 | 500.00 | 1.00 |
| ***11*** | 5 | 6 | 100 | 100 | 500.00 | 600.00 | 1.10 |
| ***12*** | 10 | 0 | 100 | 100 | 1000.00 | 0.00 | 1.00 |
| ***13*** | 10 | 0 | 100 | 100 | 1000.00 | 0.00 | 1.00 |
| ***14*** | 10 | 10 | 100 | 100 | 1000.00 | 1000.00 | 2.00 |
| ***15*** | 0 | 10 | 100 | 100 | 0.00 | 1000.00 | 1.00 |
| ***16*** | 20 | 15 | 100 | 100 | 2000.00 | 1371.42 | 3.37 |
| **Total Simulation Time:** *23.47 μs* | | | | | | | |

**Supplementary Table 11. SoPIP2:complex adaptive sampling summary.** The first round of simulation, "Round 1", consists of a single 1 $\mu$s trajectory originated from each of the crystal structure embeddings. This single trajectory took a few "rounds" to complete due to wallclock time limitations. The first "true" round of adaptive sampling is labelled as "Round 4". Each trajectory within a round of sampling outside of "Round 1" is set to be 100 ns in length.

| complex | Parallel Simulations | | Trajectory Length (ns) | | Round Simulation Time (ns) | | Aggregate Time (μs) |
|---|---|---|---|---|---|---|---|
| Round | 2b5f (open) | 1z98 (closed) | 2b5f (open) | 1z98 (closed) | 2b5f (open) | 1z98 (closed) | |
| ***1*** | 1 | 1 | 1000 | 1000 | 791.17 | 1200.00 | 1.99 |
| ***4*** | 6 | 4 | 100 | 100 | 600.00 | 400.00 | 1.00 |
| ***5*** | 10 | 10 | 60 | 100 | 664.30 | 1000.00 | 1.66 |
| ***6*** | 10 | 10 | 100 | 100 | 1000.00 | 1000.00 | 2.00 |
| ***7*** | 10 | 10 | 100 | 100 | 1000.00 | 1000.00 | 2.00 |
| ***8*** | 5 | 5 | 100 | 100 | 500.00 | 500.00 | 1.00 |
| ***9*** | 5 | 5 | 100 | 100 | 500.00 | 500.00 | 1.00 |
| ***10*** | 10 | 10 | 100 | 100 | 1000.00 | 1000.00 | 2.00 |
| ***11*** | 10 | 10 | 100 | 100 | 1000.00 | 1000.00 | 2.00 |
| ***12*** | 10 | 10 | 100 | 100 | 1000.00 | 1000.00 | 2.00 |
| ***13*** | 10 | 10 | 100 | 100 | 1000.00 | 1000.00 | 2.00 |
| ***14*** | 10 | 10 | 100 | 100 | 1000.00 | 1000.00 | 2.00 |
| ***15*** | 10 | 10 | 100 | 100 | 1000.00 | 1000.00 | 2.00 |
| ***16*** | 10 | 10 | 100 | 100 | 1000.00 | 1000.00 | 2.00 |
| ***17*** | 10 | 10 | 100 | 100 | 1000.00 | 1000.00 | 2.00 |
| ***18*** | 10 | 10 | 100 | 100 | 1000.00 | 1000.00 | 2.00 |
| ***19*** | 10 | 10 | 100 | 100 | 1000.00 | 1000.00 | 2.00 |
| ***20*** | 10 | 10 | 100 | 100 | 1000.00 | 1000.00 | 2.00 |
| ***21*** | 0 | 10 | 100 | 100 | 0.00 | 1000.00 | 1.00 |
| ***22*** | 0 | 10 | 100 | 100 | 0.00 | 961.39 | 0.96 |
| ***23*** | 10 | 10 | 100 | 100 | 1000.00 | 1000.00 | 2.00 |
| ***24*** | 15 | 15 | 100 | 100 | 1500.00 | 1500.00 | 3.00 |
| ***25*** | 10 | 10 | 100 | 100 | 1000.00 | 1000.00 | 2.00 |
| **Total Simulation Time:** *41.62 μs* | | | | | | | |

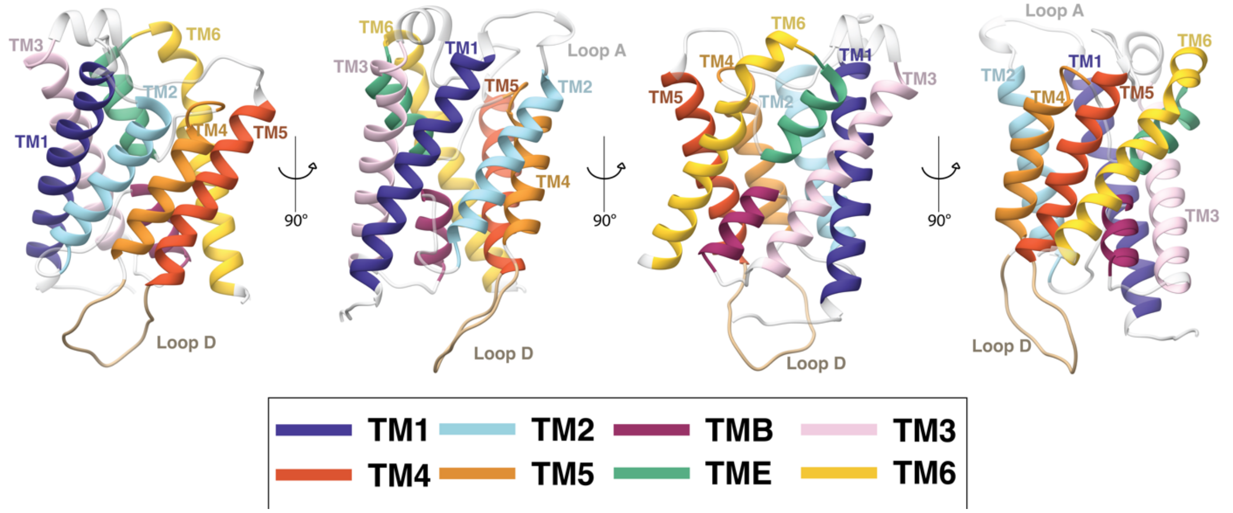


**Supplementary Figure 1. SoPIP2;1 topology.** The helices and loop D are colored and labelled on the SoPIP2;1 open macrostate. Loop A is labelled when visible. TM indicates transmembrane domain according to the aquaporin topology. TMB and TME are two half helices in the lower leaflet and upper leaflet, respectively, of the lipid bilayer.

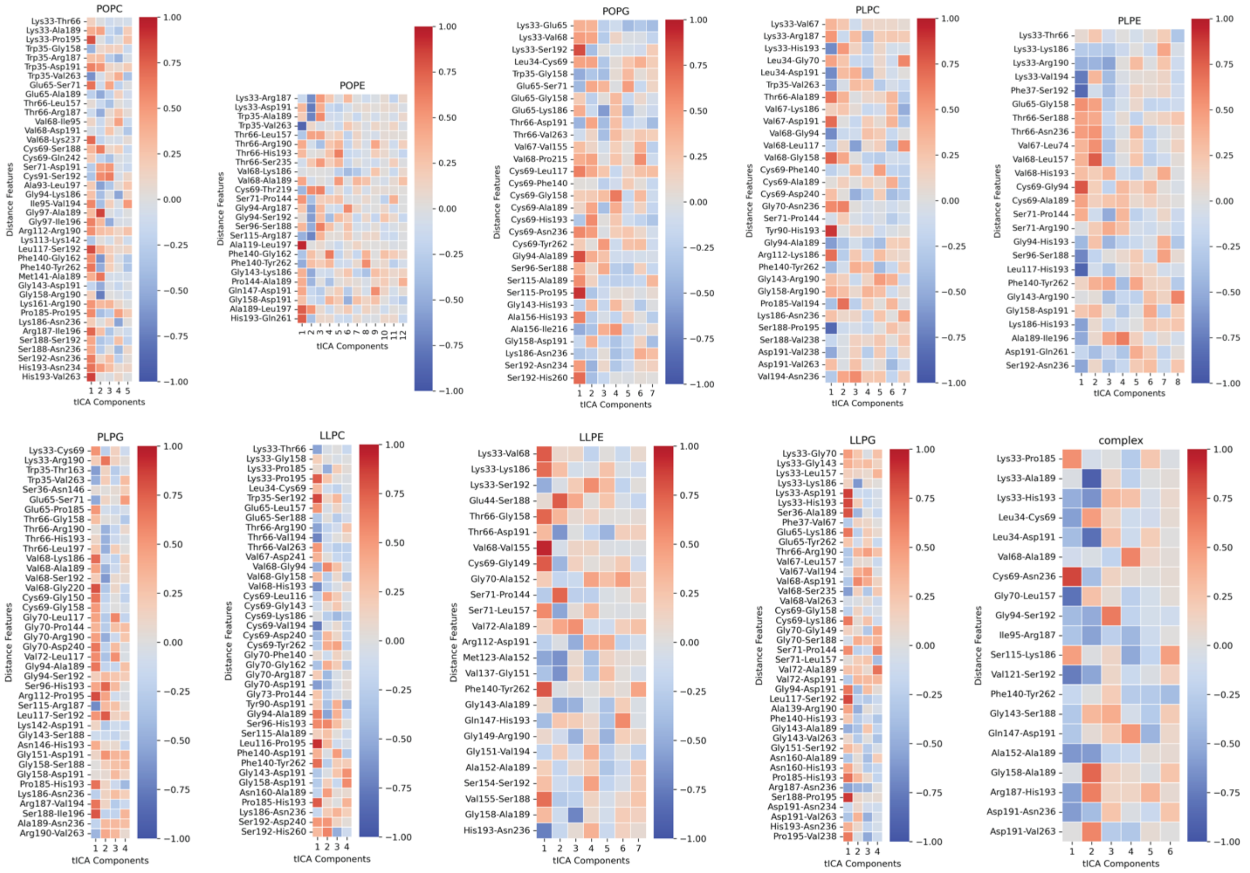


**Supplementary Figure 2. Distance features and their correlation to tICA components generated by RRCS and spectral oASIS workflow.** All heatmaps represent correlations for features used in each of the final SoPIP2;1 MSMs.

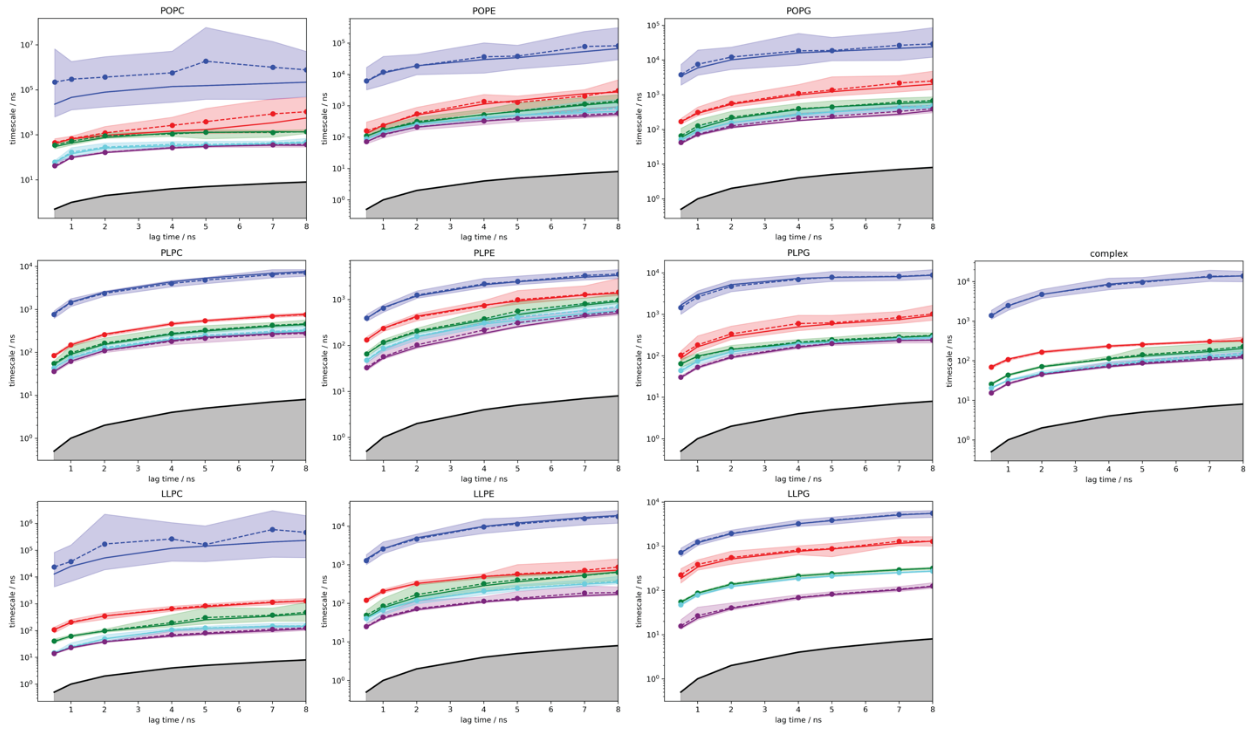


**Supplementary Figure 3. Implied timescale plots for the SoPIP2:bilayer MSMs capturing the transitions for loop D conformational change.** Implied timescale plots were calculated with Bayesian error.

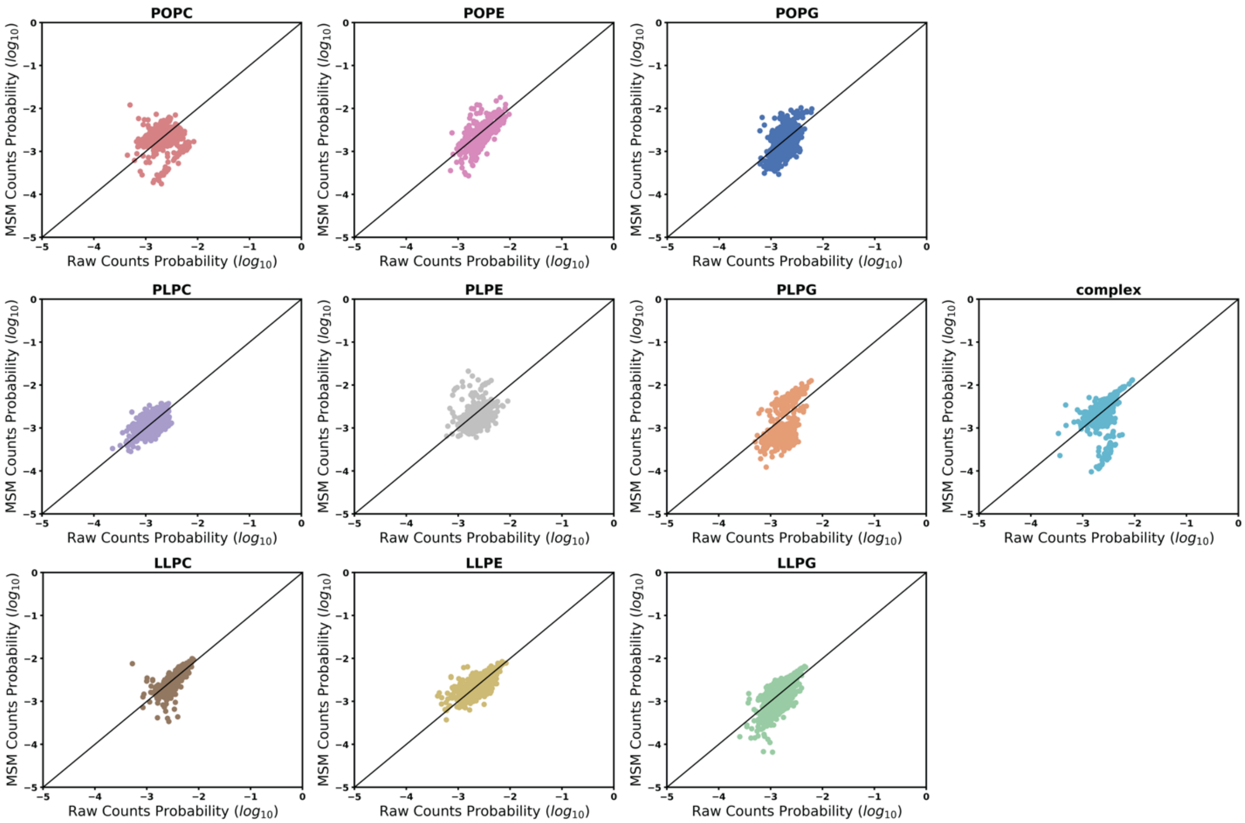


**Supplementary Figure 4. Raw counts versus MSM population for each microstate cluster of SoPIP2:bilayer conformational dynamics.**

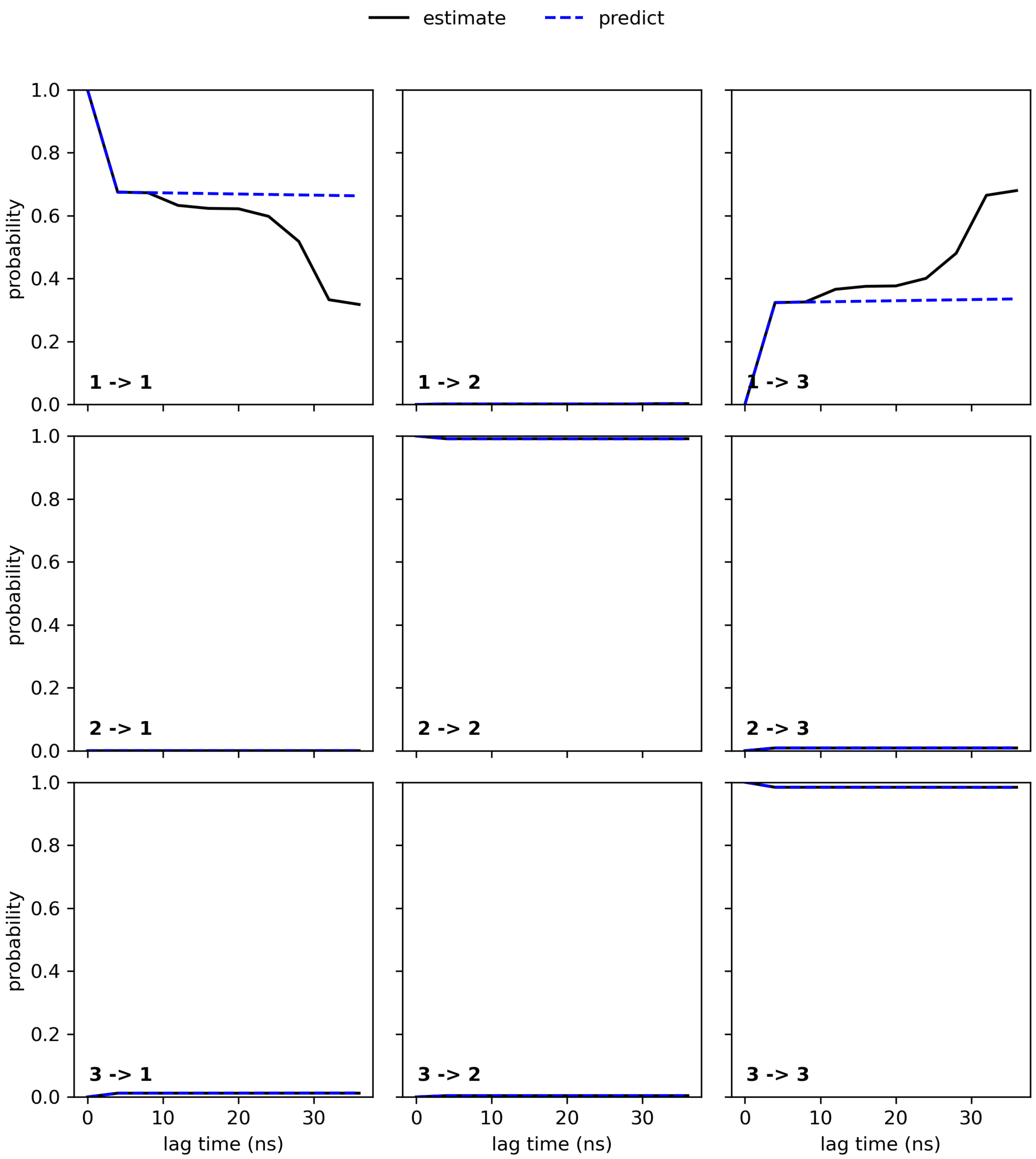


**Supplementary Figure 5. Chapman–Kolmogorov test for the MSM of SoPIP2:POPC conformational dynamics.**

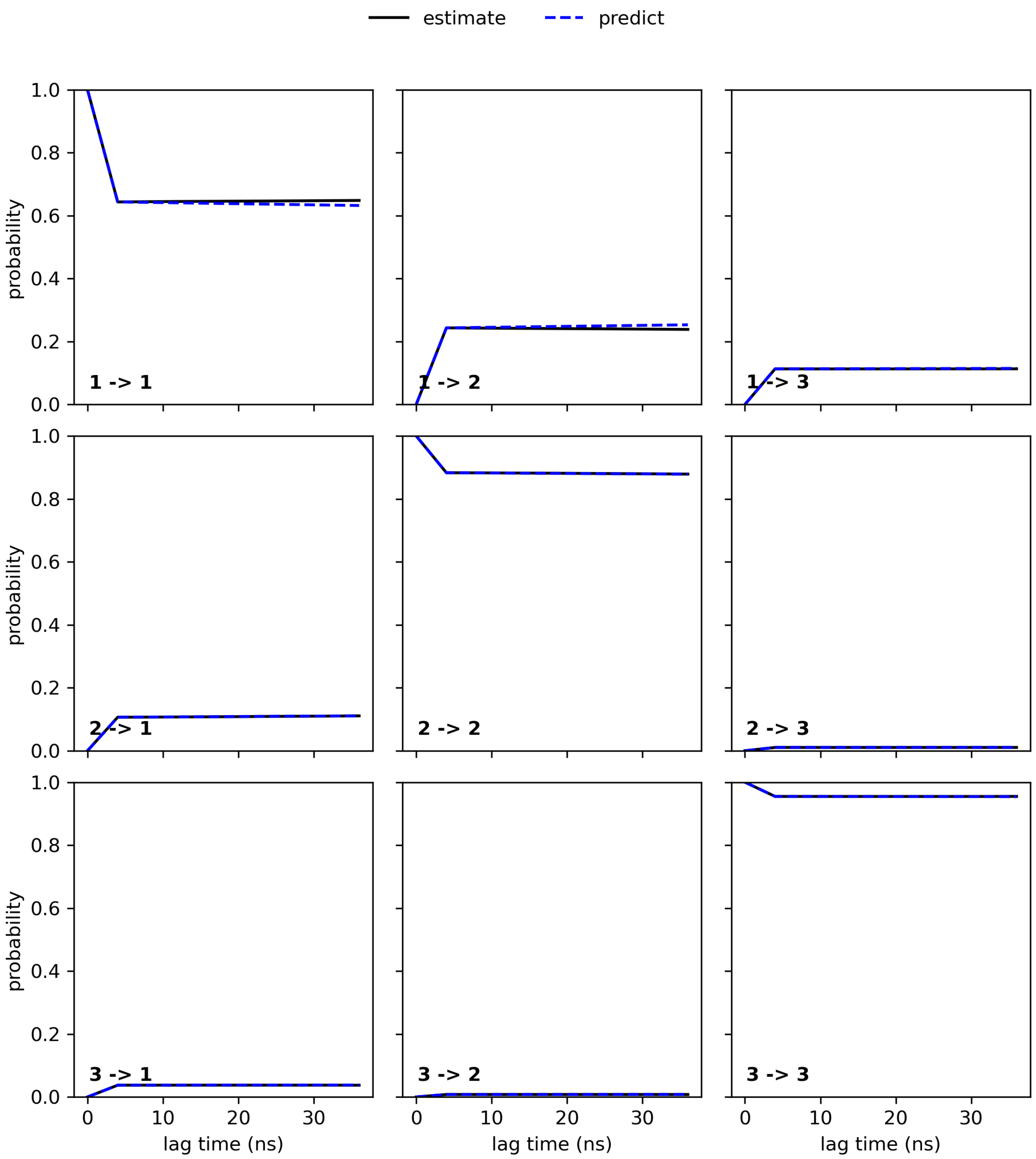


**Supplementary Figure 6. Chapman–Kolmogorov test for the MSM of SoPIP2:POPE conformational dynamics.**

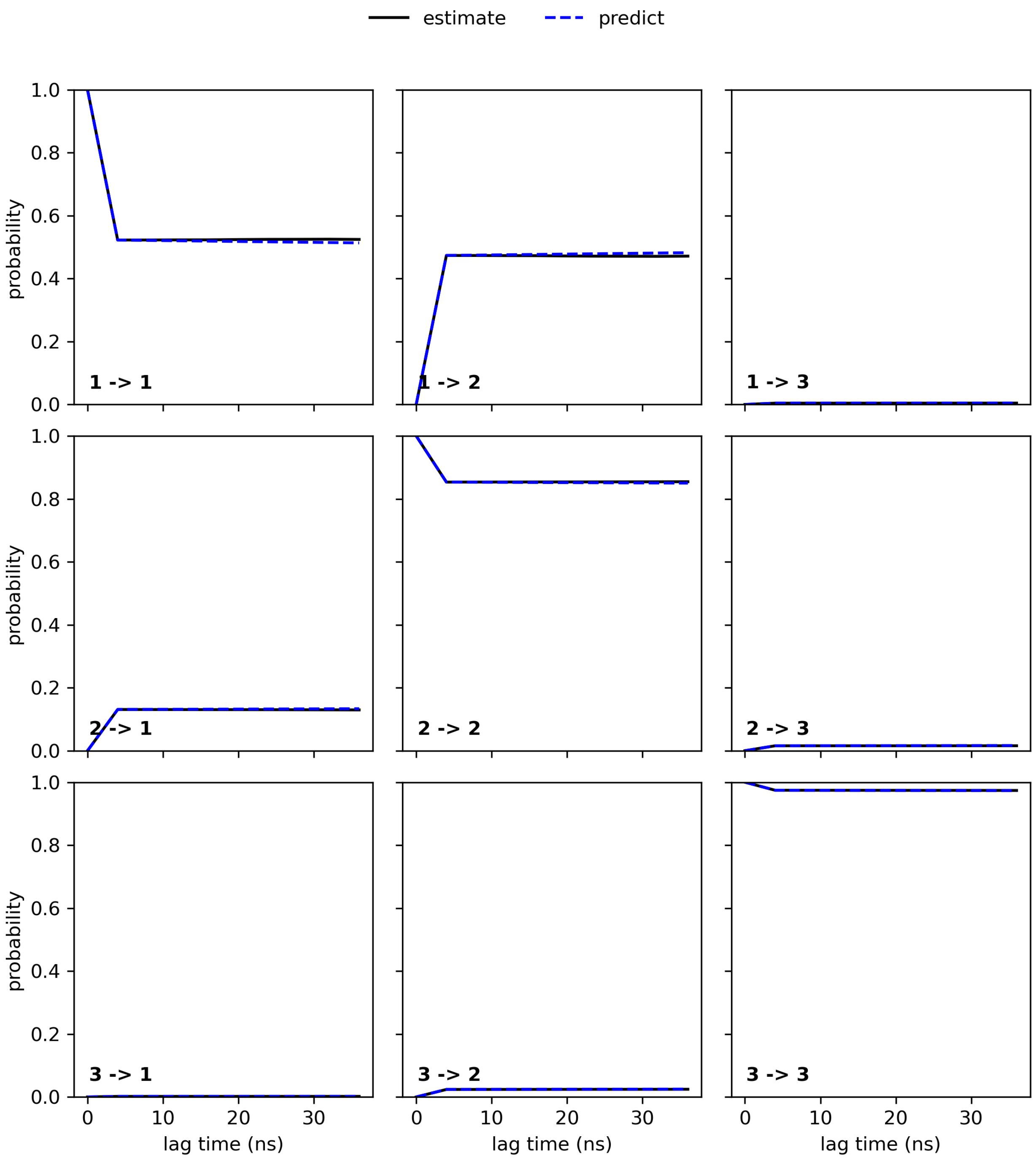


**Supplementary Figure 7. Chapman–Kolmogorov test for the MSM of SoPIP2:POPG conformational dynamics.**

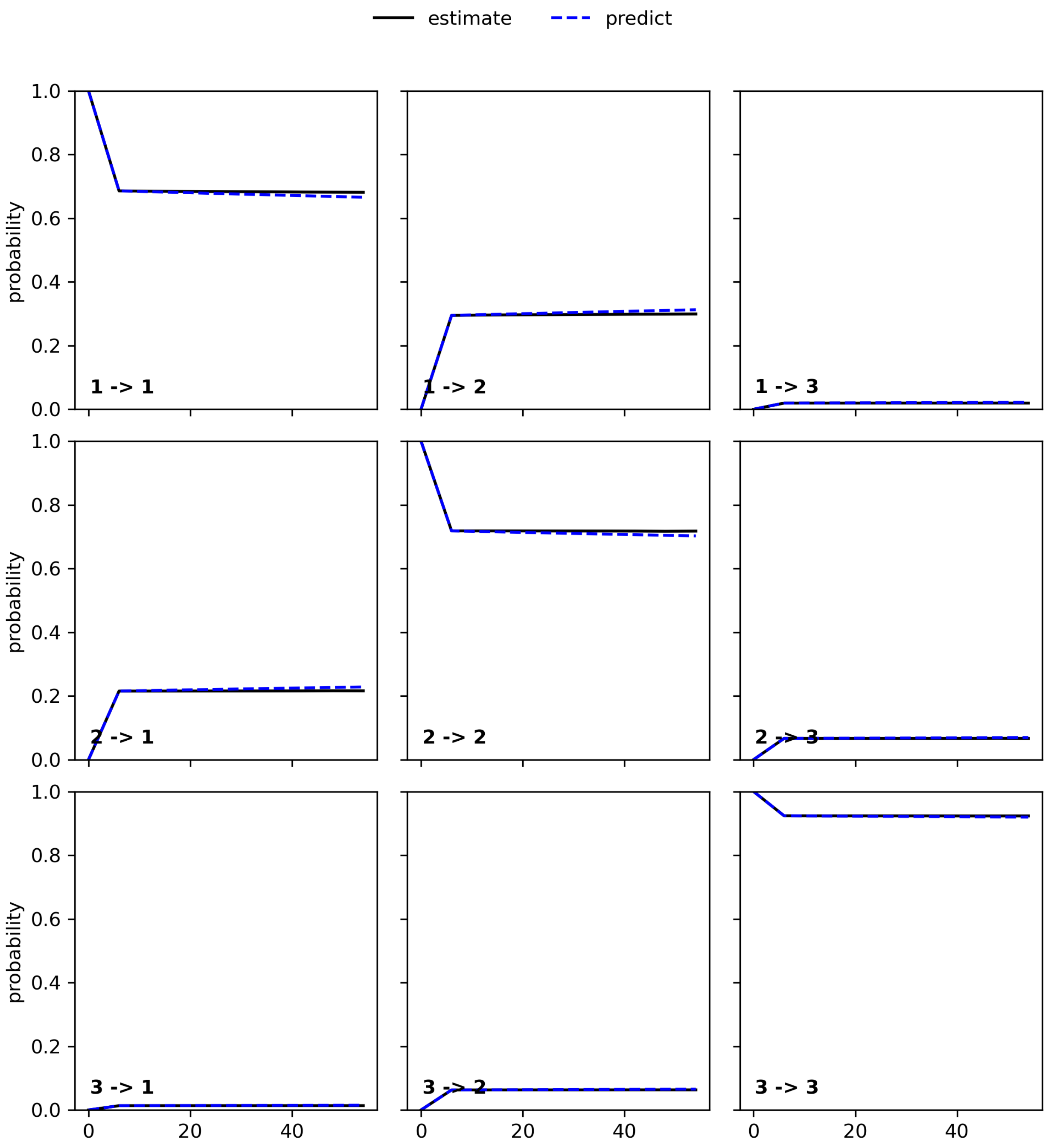


**Supplementary Figure 8. Chapman–Kolmogorov test for the MSM of SoPIP2:PLPC conformational dynamics.**

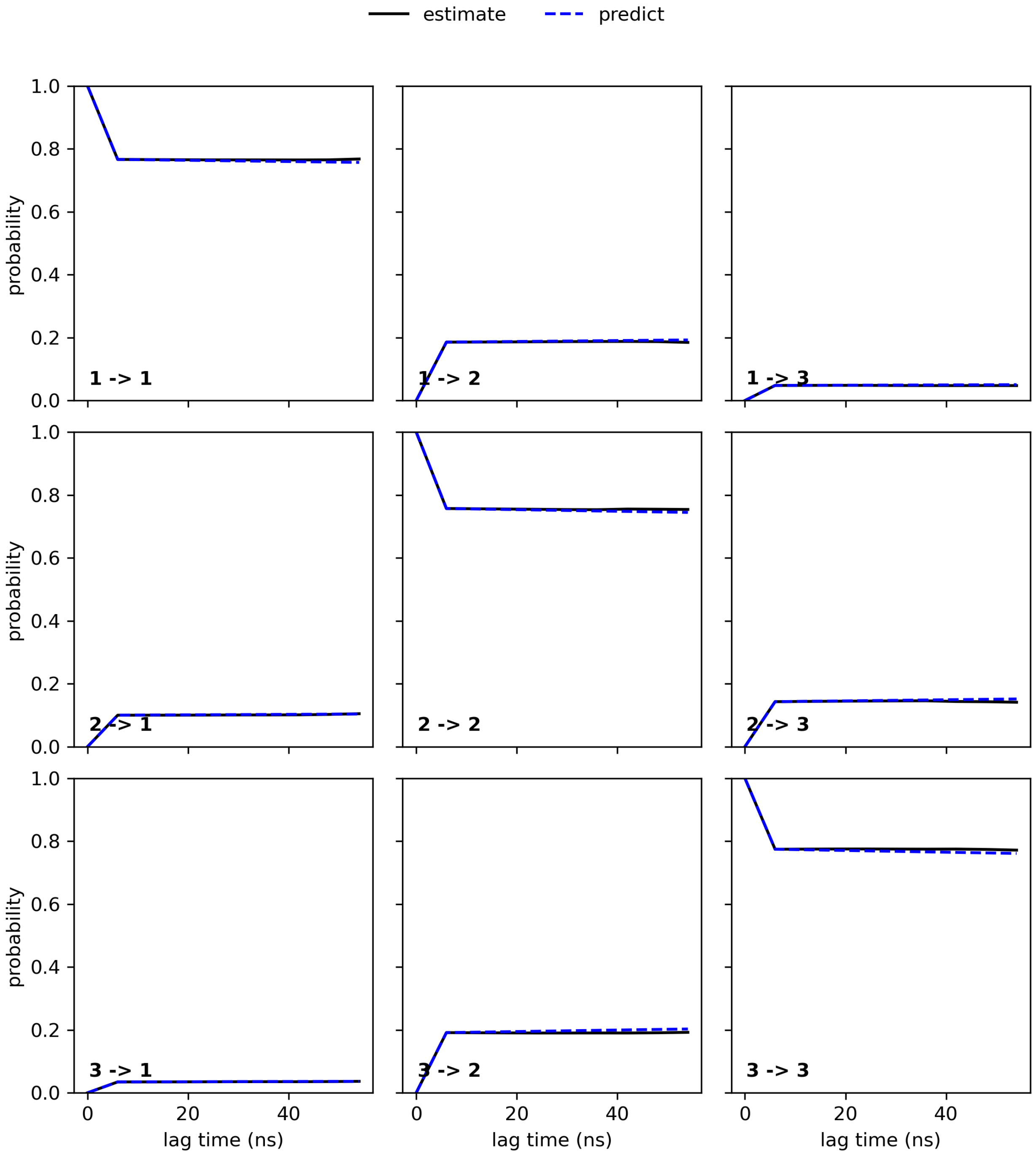


**Supplementary Figure 9. Chapman–Kolmogorov test for the MSM of SoPIP2:PLPE conformational dynamics.**

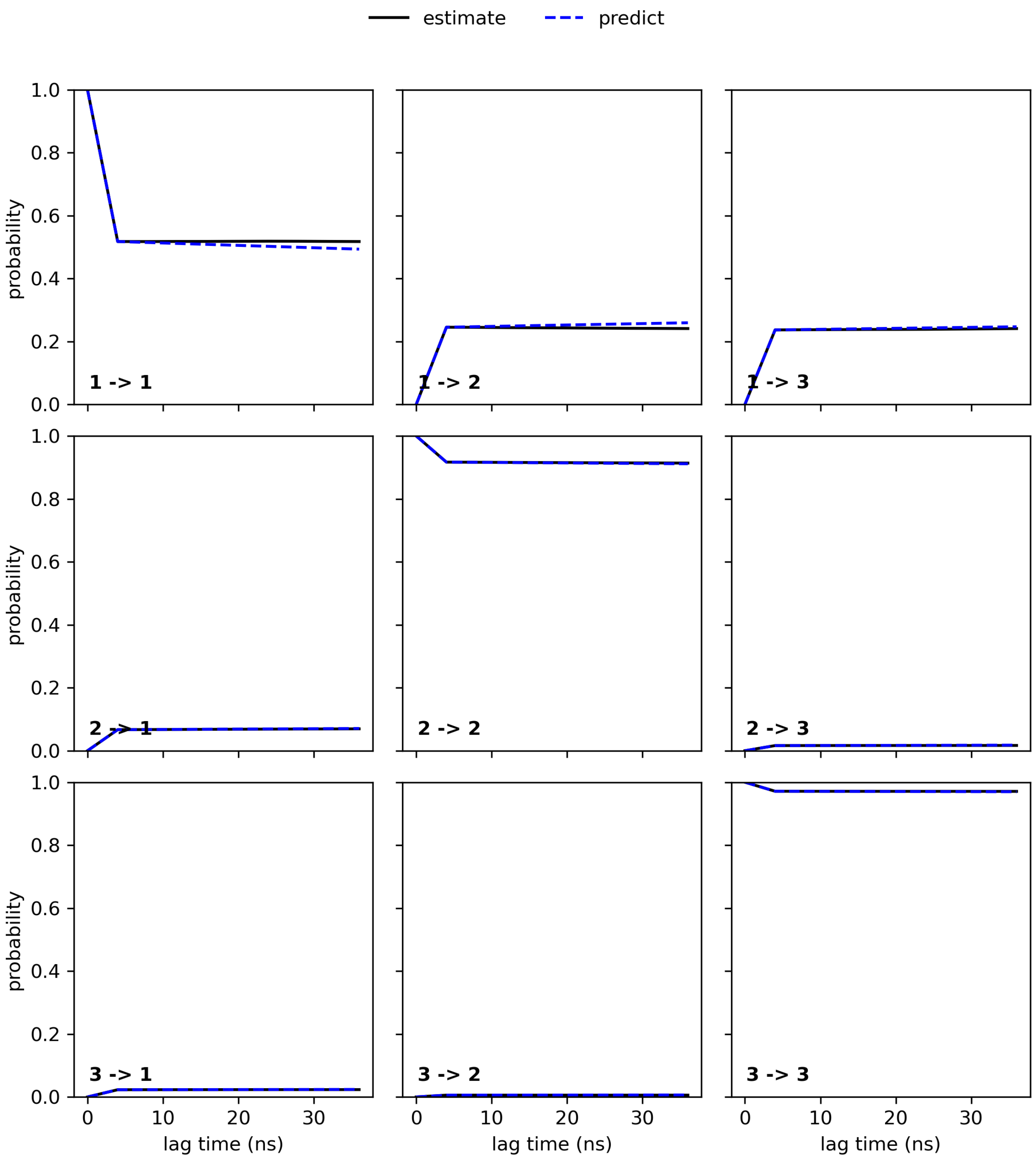


**Supplementary Figure 10. Chapman–Kolmogorov test for the MSM of SoPIP2:PLPG conformational dynamics.**

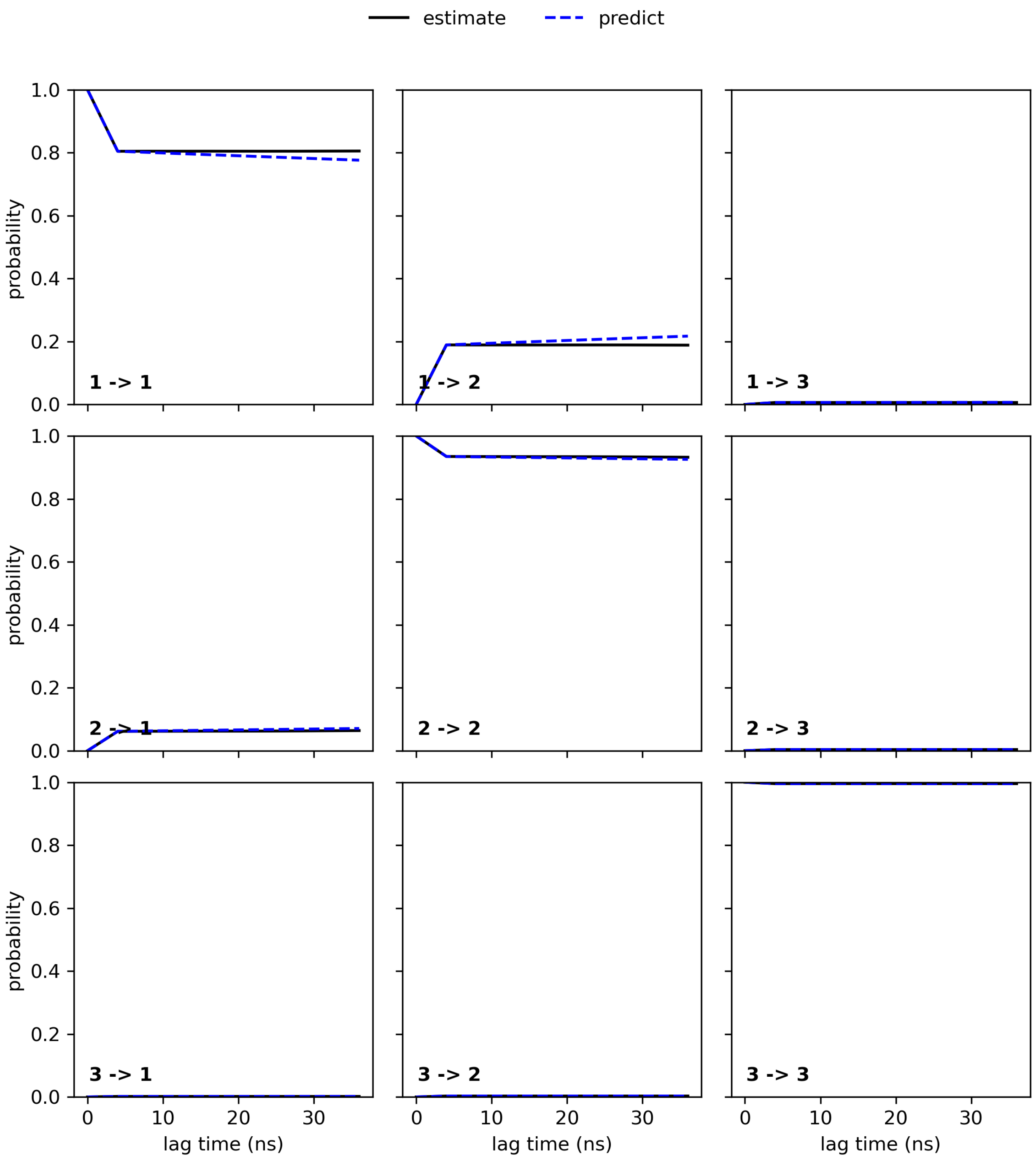


**Supplementary Figure 11. Chapman–Kolmogorov test for the MSM of SoPIP2:LLPC conformational dynamics.**

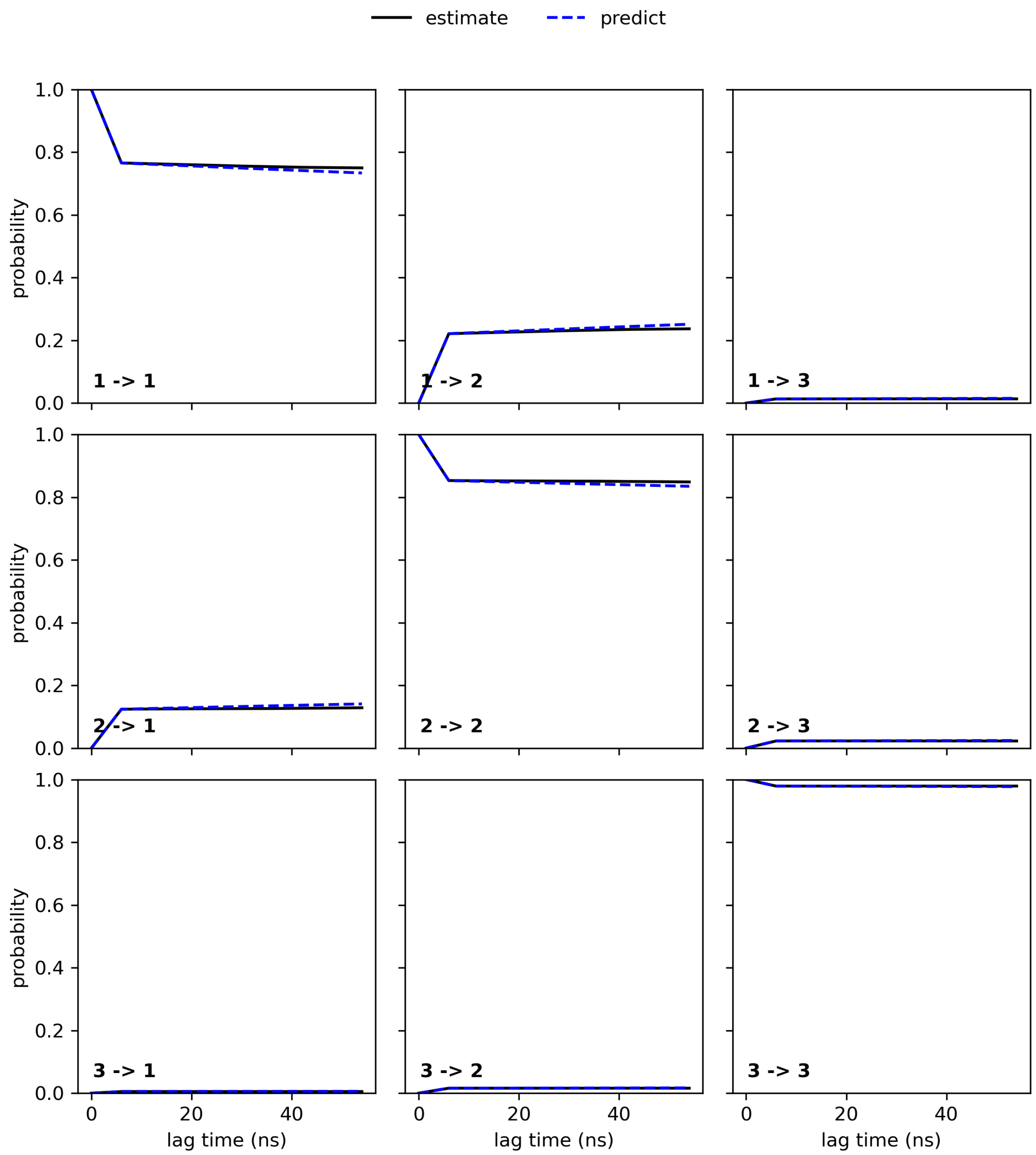


**Supplementary Figure 12. Chapman–Kolmogorov test for the MSM of SoPIP2:LLPE conformational dynamics.**

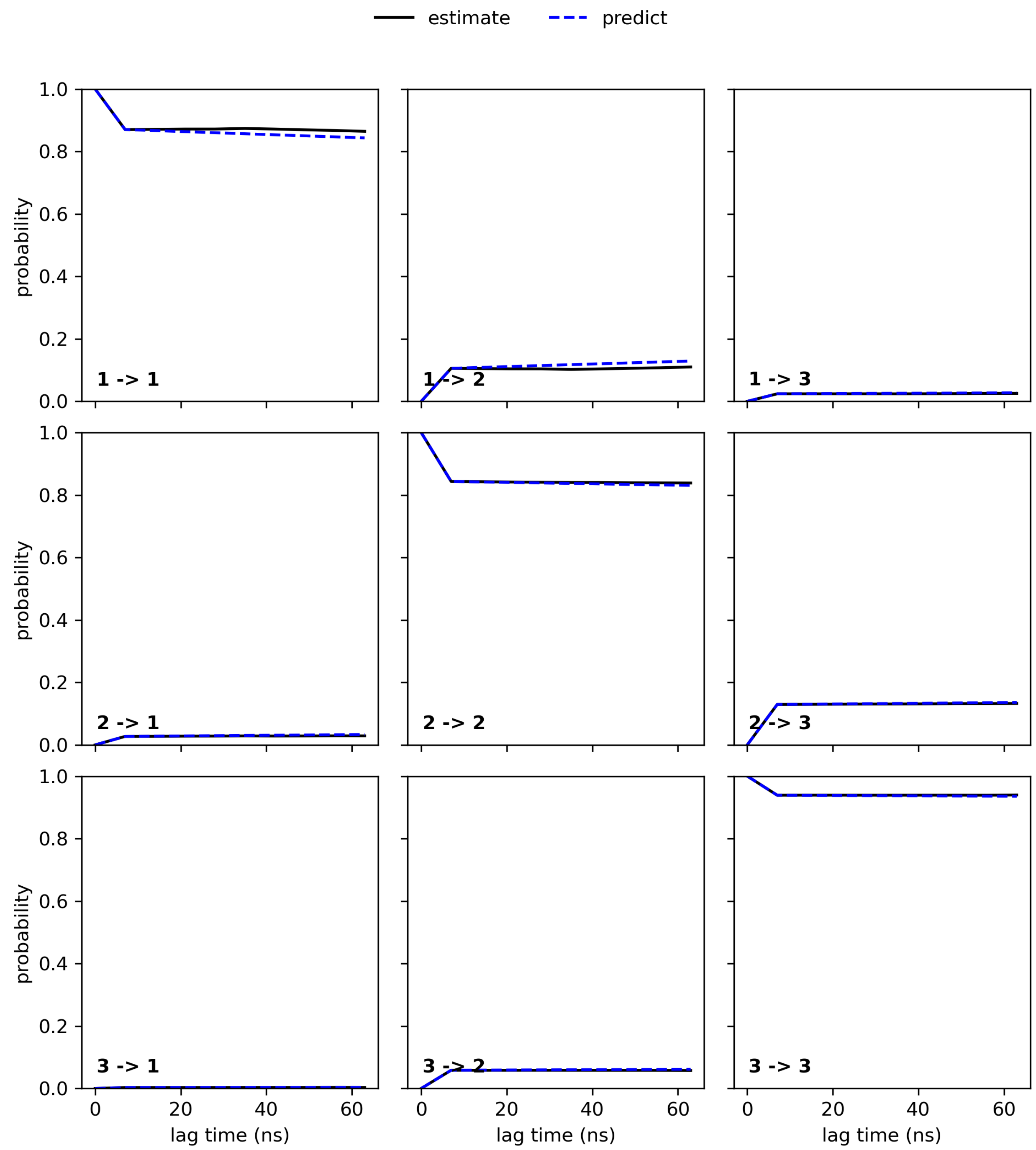


**Supplementary Figure 13. Chapman–Kolmogorov test for the MSM of SoPIP2:LLPG conformational dynamics.**

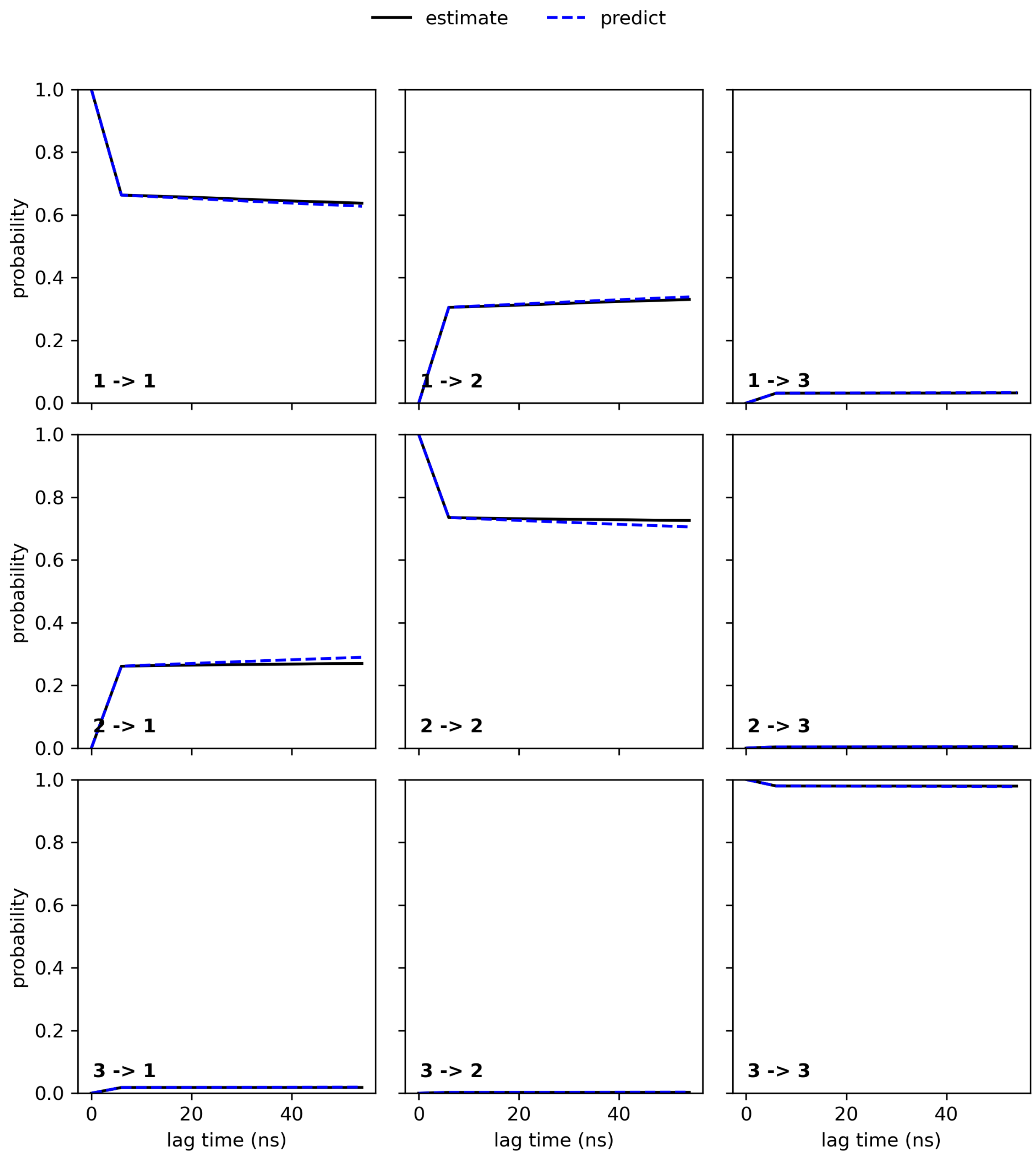


**Supplementary Figure 14. Chapman–Kolmogorov test for the MSM of SoPIP2:complex conformational dynamics.**

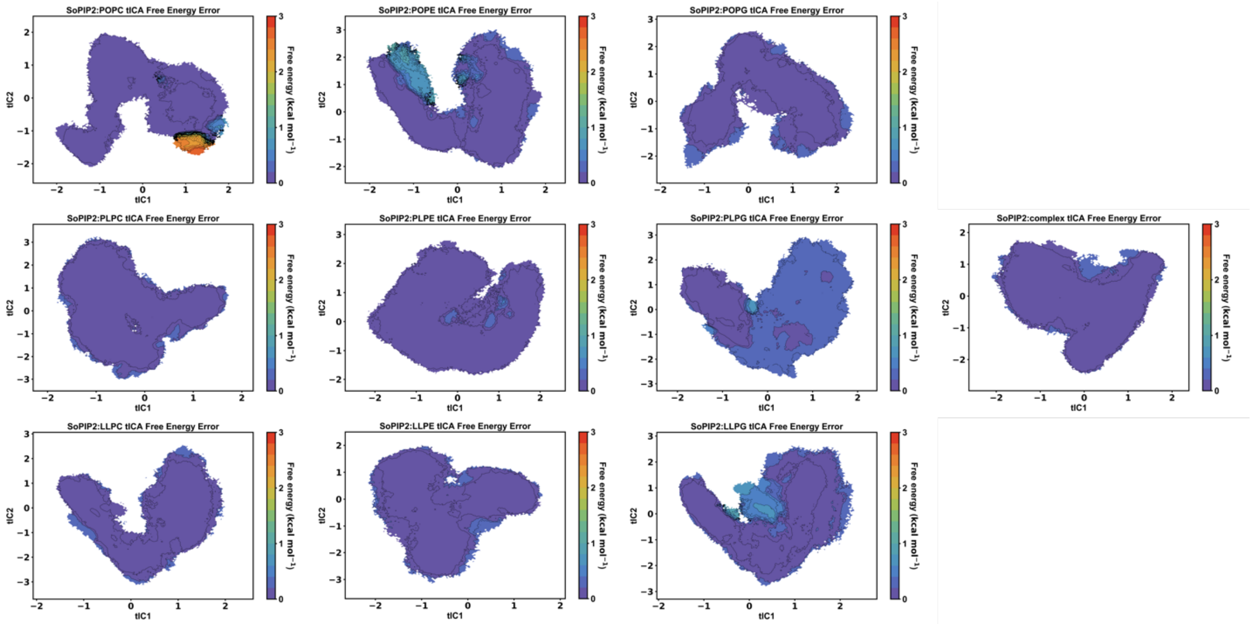


**Supplementary Figure 15. Free energy error from the 200-sample bootstrapping protocol for each SoPIP2:bilayer system.**

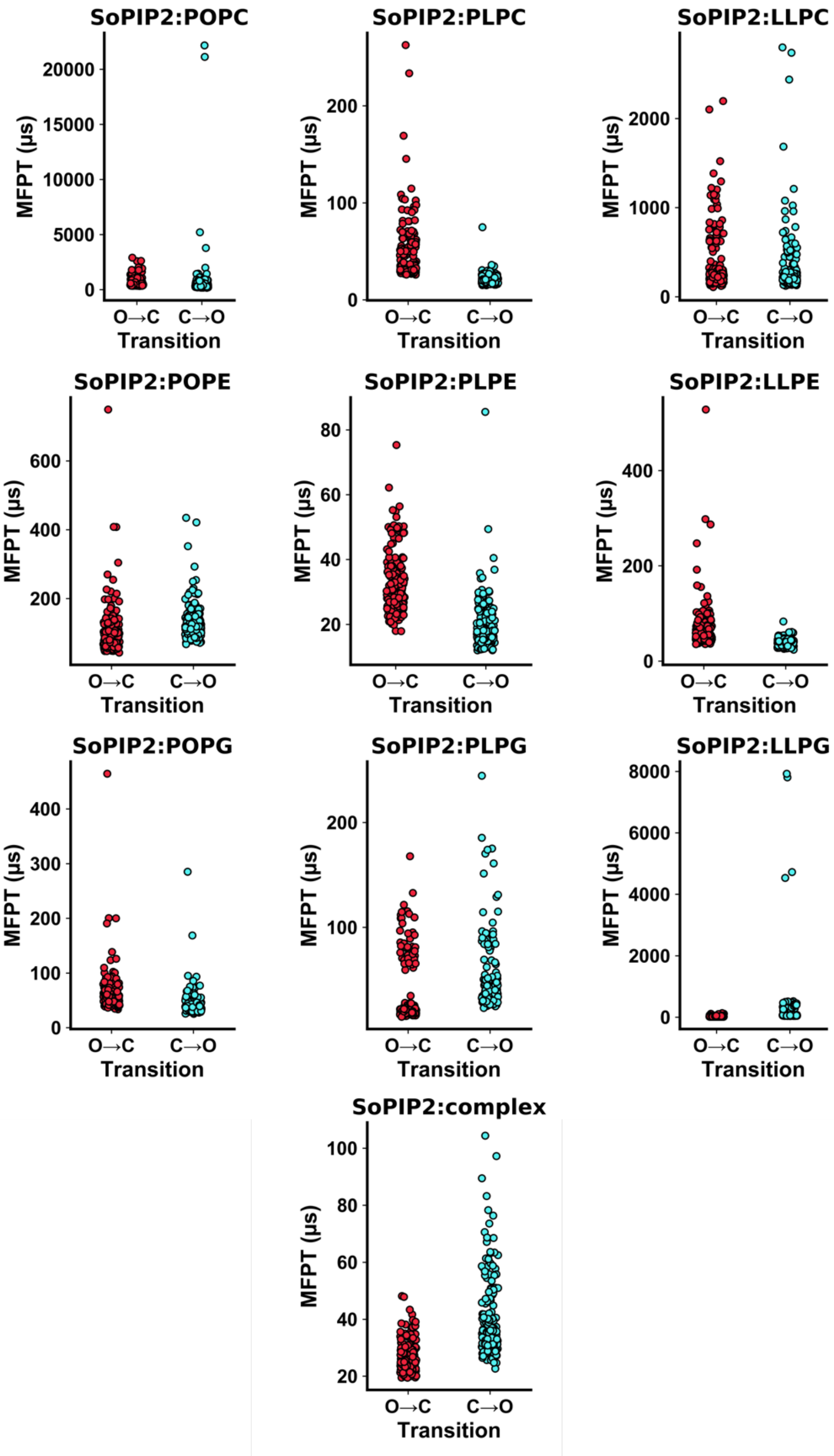


**Supplementary Figure 16. Data distributions for MFPT calculations of open-to-closed (O → C) and closed-to-open (O → C) transitions.**

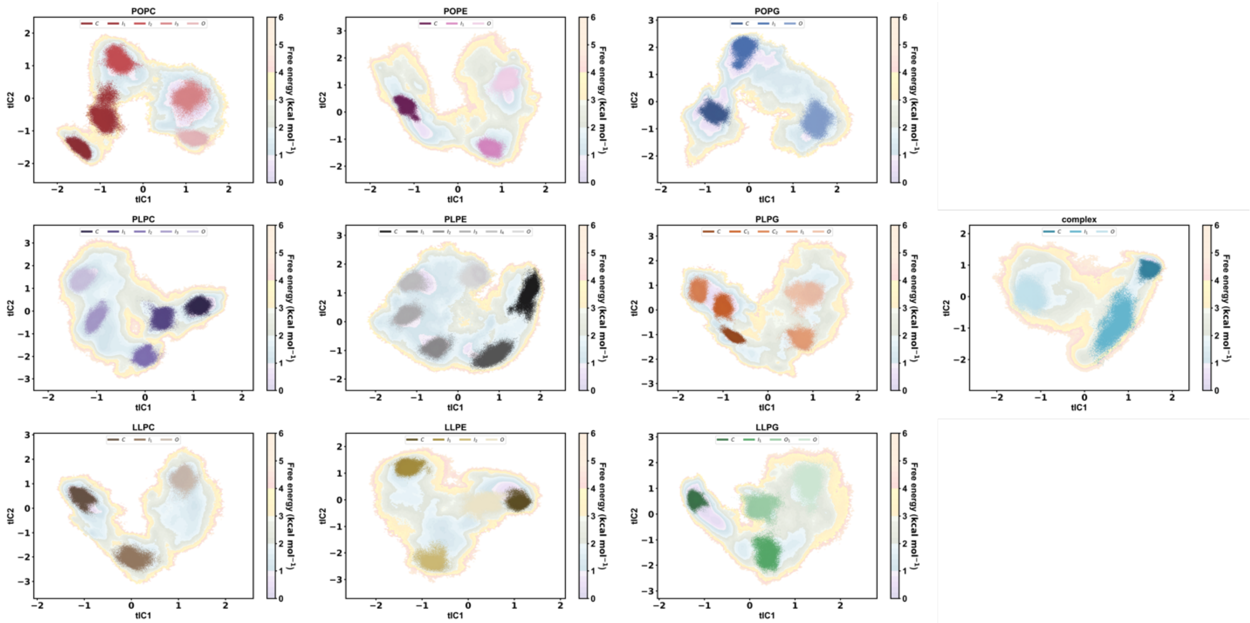


**Supplementary Figure 17. Selected continuous trajectories for the water transport analysis based on tICA macrostate identification.**

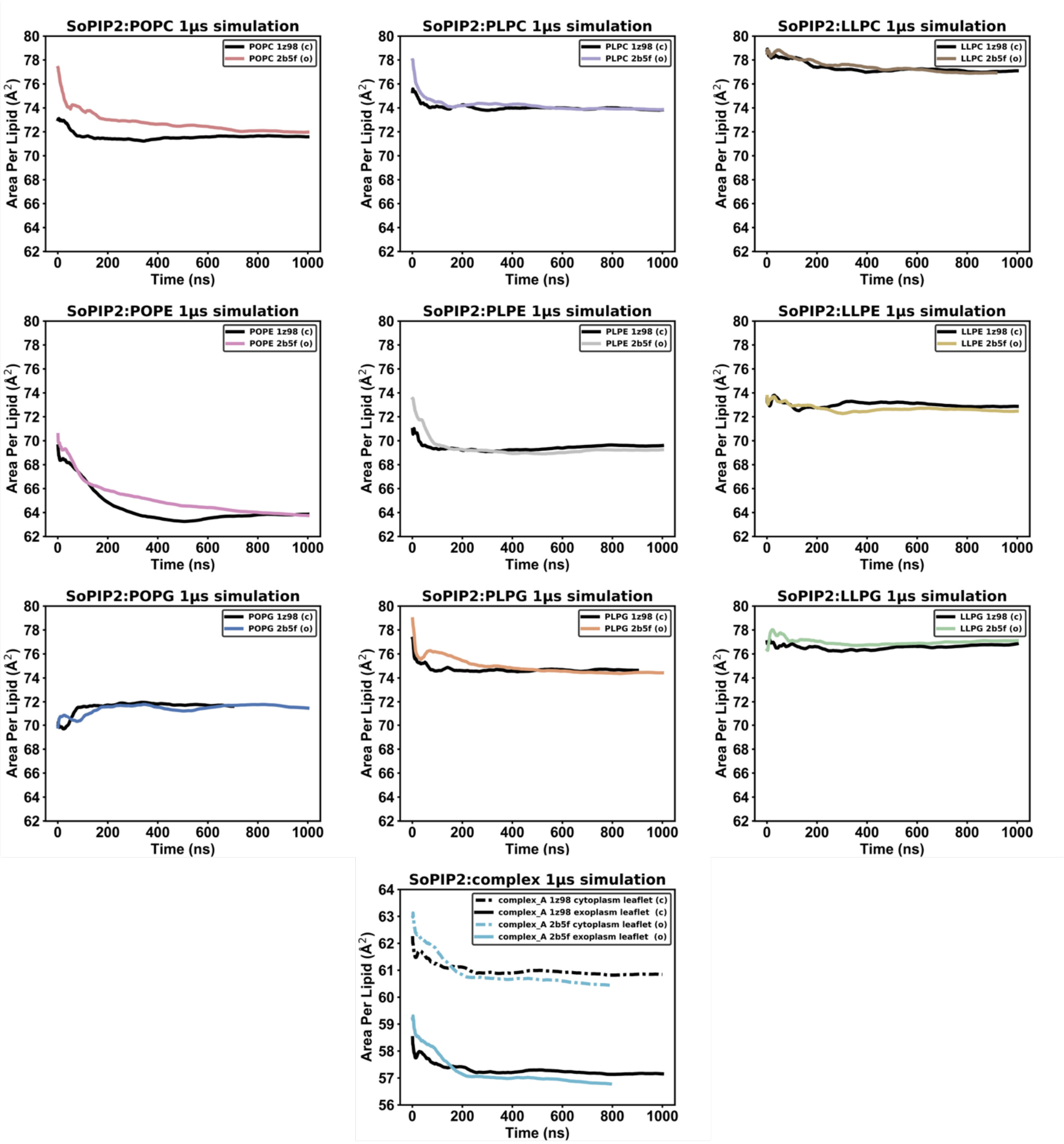


**Supplementary Figure 18. Area per lipid (APL) cumulative average from each initial 1$\mu$s production run.**

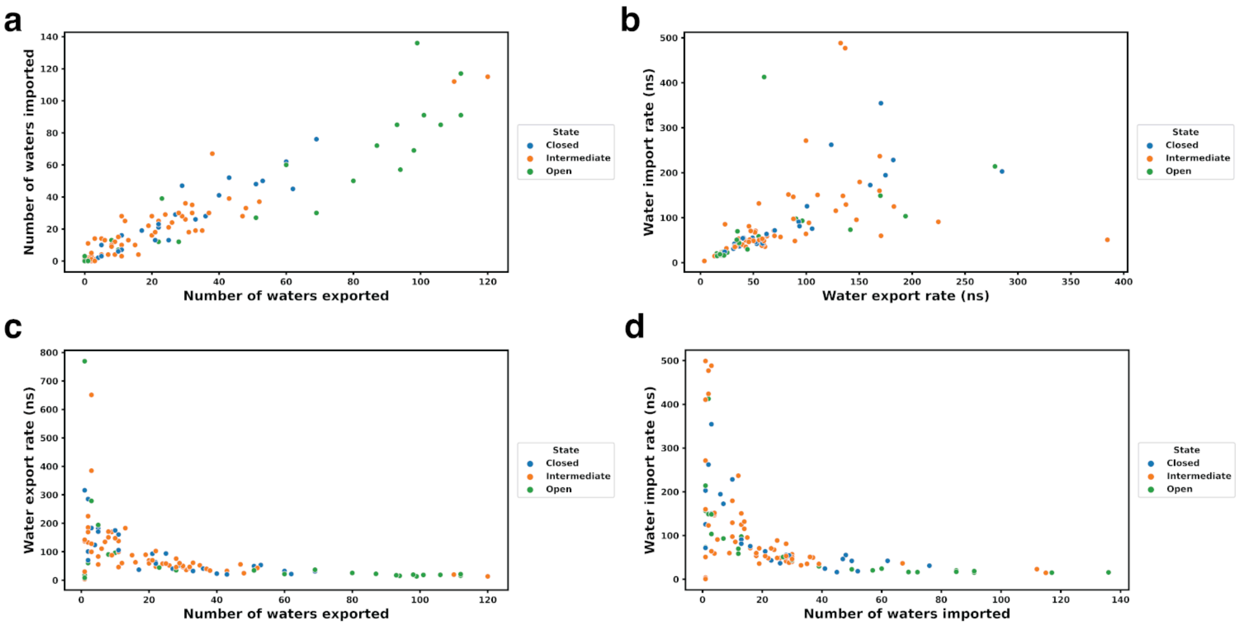


**Supplementary Figure 19. Relationship between number of waters transported and the rate of transport.** (**a**) The positive correlation between the number of waters transported inside (imported into) the pore versus the number of waters transported outside (exported from) the pore. (**b**) Slight correlation between the export and import rate of transported waters. (**c**) Relationship between the rate and number of waters for export processes. (**d**) Relationship between the rate and number of waters for import processes.

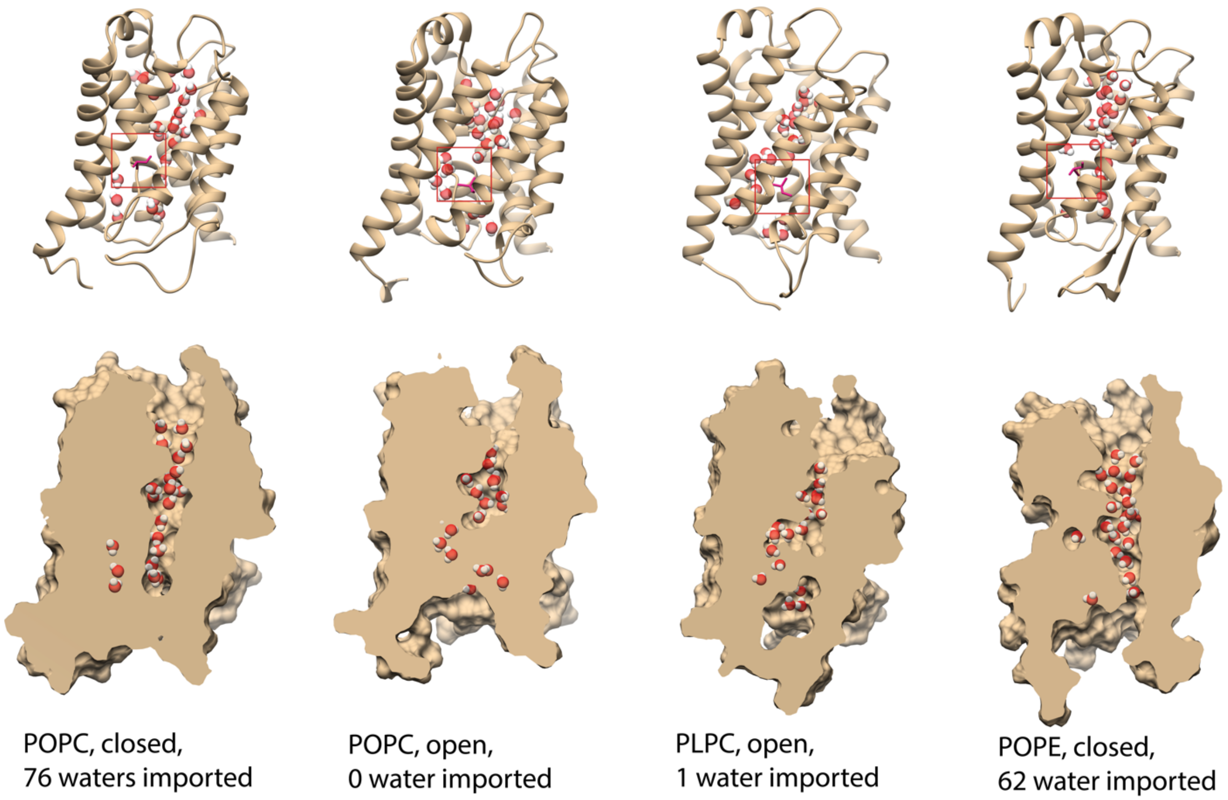


**Supplementary Figure 20. Pore cavity structure with respect to loop D conformation throughout different types of example transport cases.**

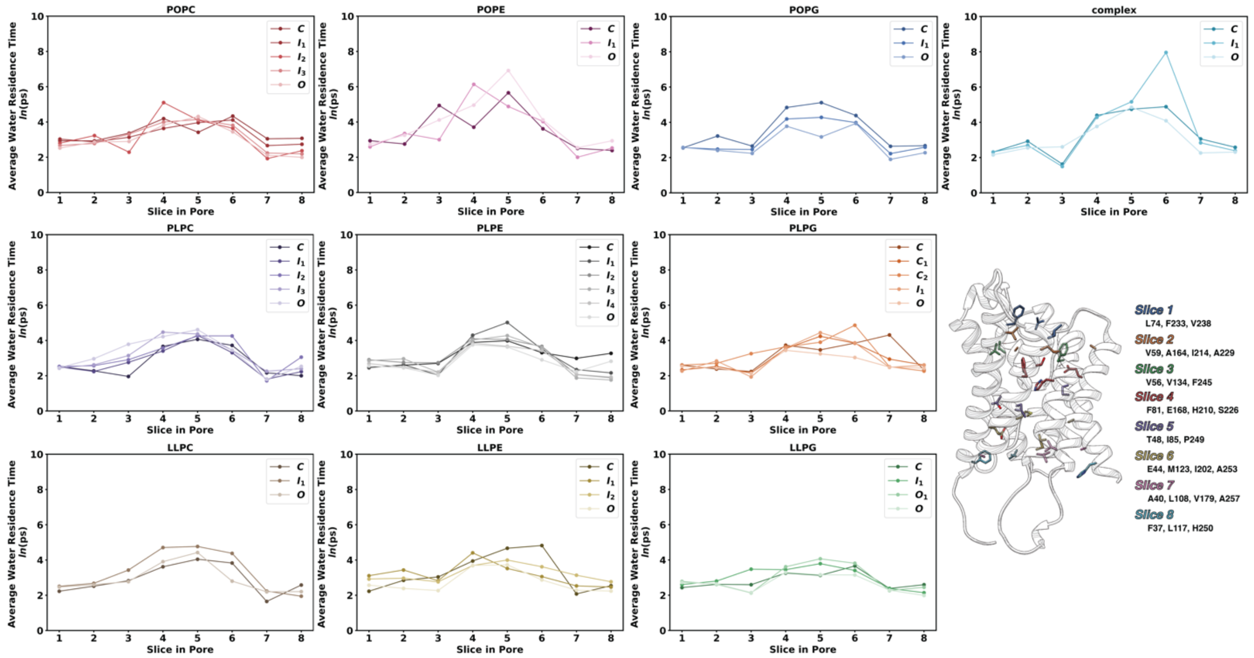


**Supplementary Figure 21. Residence time of water molecules at each slice of the SoPIP2;1 pore.** The protein is separated into eight lateral slices along the vertical z-vector of the central pore. Each slice is defined as a cylinder whose center is given by the center of geometry from the $C_\alpha$ of 3-4 residues shown in the snapshot. The height of the cylinder is ± 2 Å from the cylinder center. The radius of the cylinder is 8 Å. The selection is done dynamically with MDAnalysis 2.0.0 "cyzone" geometric atom selection. The time for which each water molecule spends continuously in a given slice is averaged for all waters found in the slice throughout the trajectory to give the residence time. Most of the residence time landscape follows a similar pattern: water molecules (1) enter in a disordered manner and spend around ~9 ps in Slices 1-2; (2) spend less amount of time in Slice 3 (the selectivity region); then (3) remain in Slices 4-6 for a significantly higher time of ~65-80 ps (the NPA region and pore center); and finally (4) spend ~6 ps in the intracellular regions of the channel. Deviations from this pattern include all macrostates of SoPIP2:POPE and the intermediate state of SoPIP2:complex. Reasons for the intermediate state of SoPIP2 in the complex membrane could be a water molecule stuck in the opening of the second pathway. Meanwhile, the POPE bilayer induces a general difficulty in transport due to its high acyl chain order parameters and thickness (Supplementary Figures 29-31).

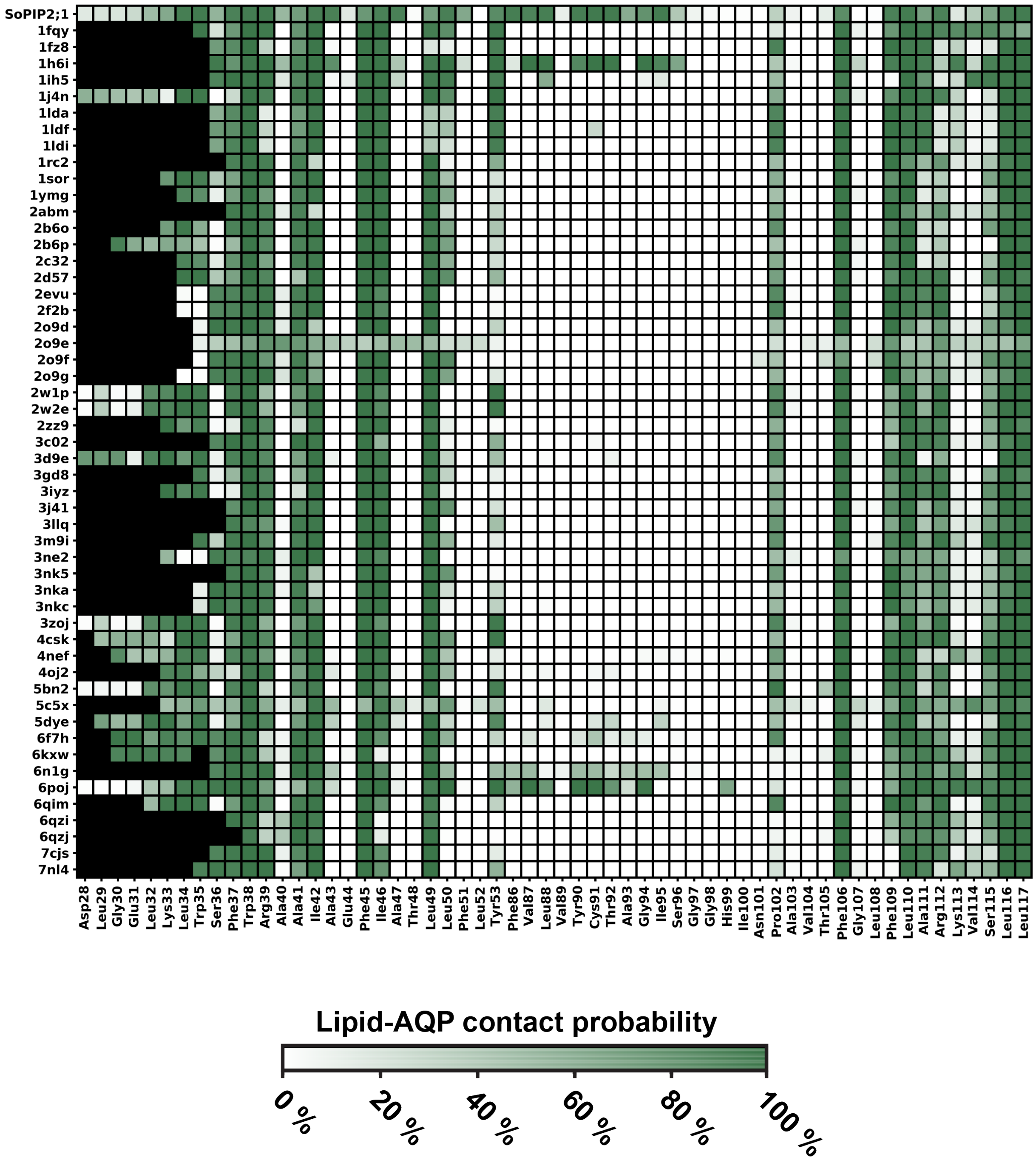


**Supplementary Figure 22. Comparison of average SoPIP2;1 lipid contact probability along the protein-lipid interface against MemProtMD simulated aquaporins (Part 1).** The colorbar reports lipid contact probability as a percentage. Black squares in the heatmap indicate aquaporin residues which did not structurally align to any portion of SoPIP2;1 with a one-to-one mapping.

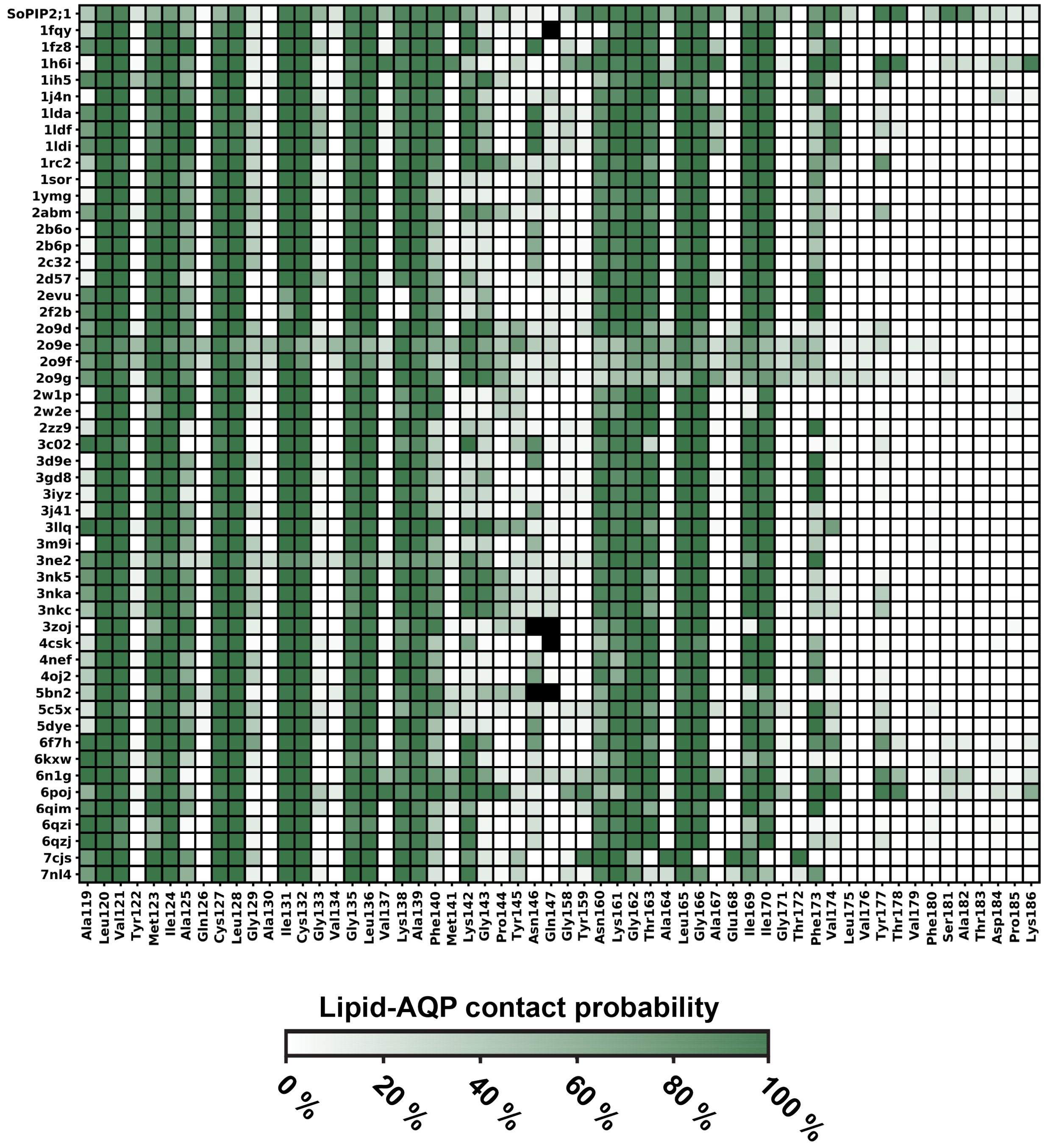


**Supplementary Figure 23. Comparison of average SoPIP2;1 lipid contact probability along the protein-lipid interface against MemProtMD simulated aquaporins (Part 2).** The colorbar reports lipid contact probability as a percentage. Black squares in the heatmap indicate aquaporin residues which did not structurally align to any portion of SoPIP2;1 with a one-to-one mapping.

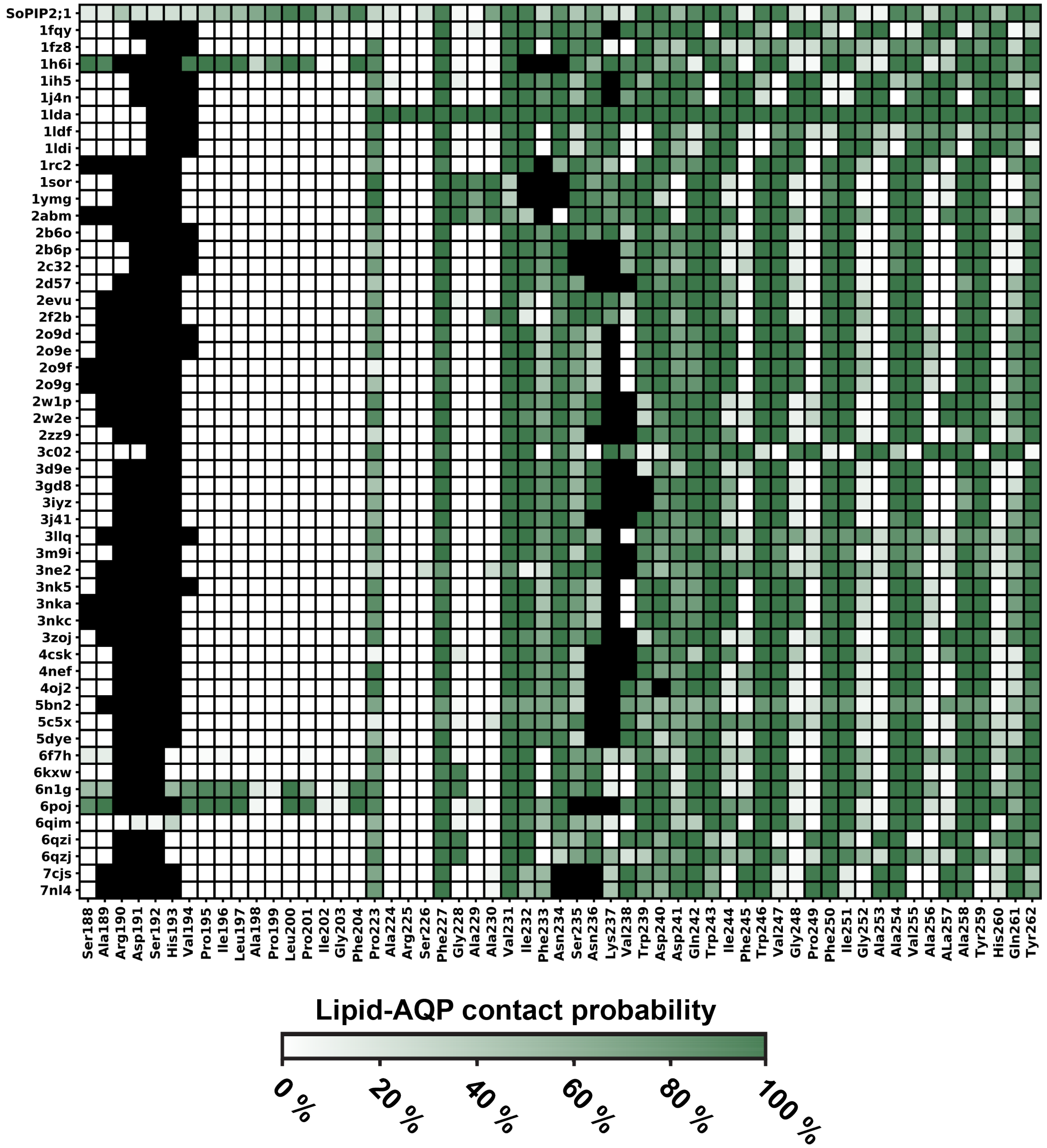


**Supplementary Figure 24. Comparison of average SoPIP2;1 lipid contact probability along the protein-lipid interface against MemProtMD simulated aquaporins (Part 3).** The colorbar reports lipid contact probability as a percentage. Black squares in the heatmap indicate aquaporin residues which did not structurally align to any portion of SoPIP2;1 with a one-to-one mapping.

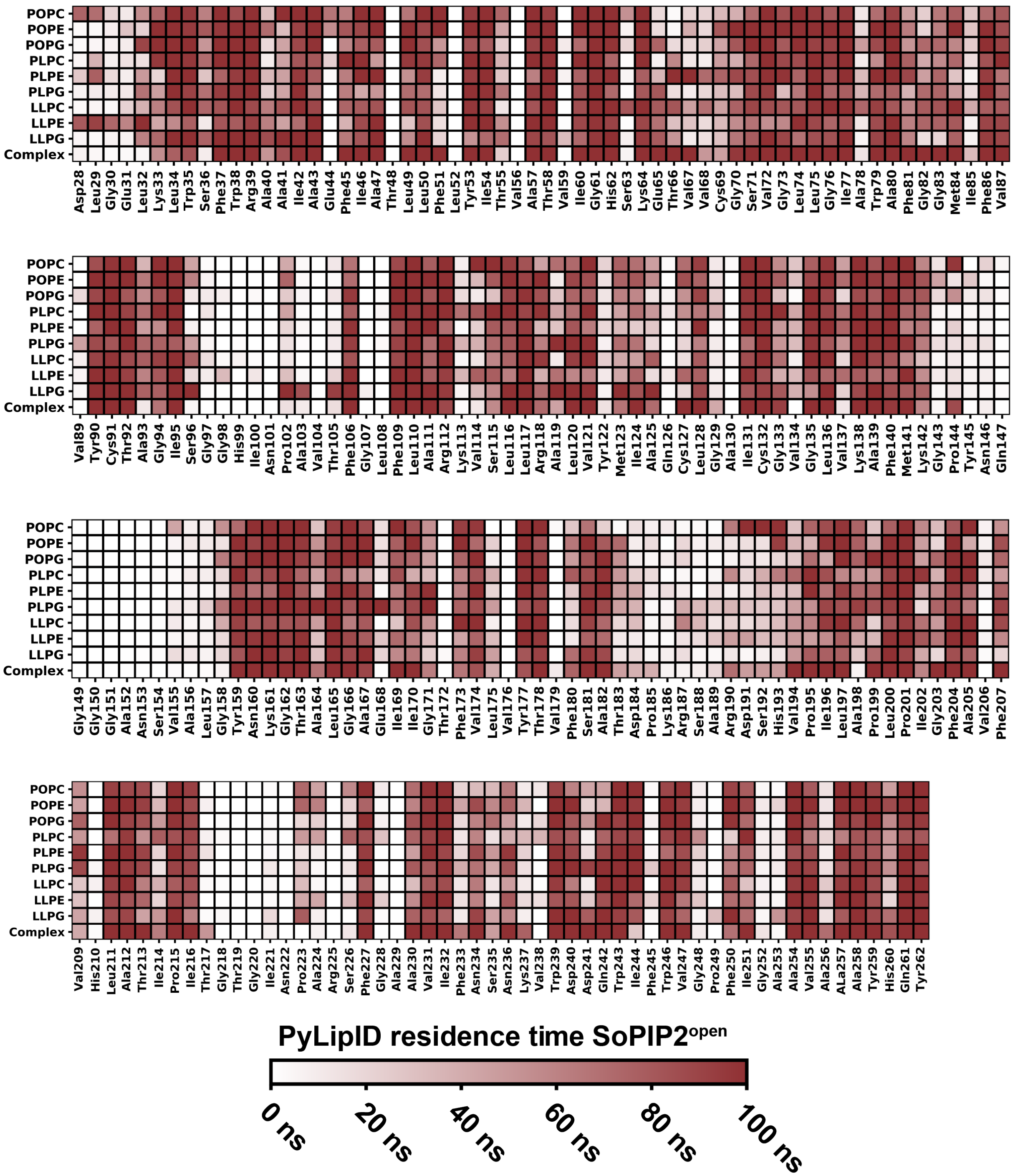


**Supplementary Figure 25. PyLipID average residence time for SoPIP2:lipid interactions along the open state.** The colorbar reports lipid residence time in nanoseconds based off calculations performed on continuous trajectories representing metastable states.

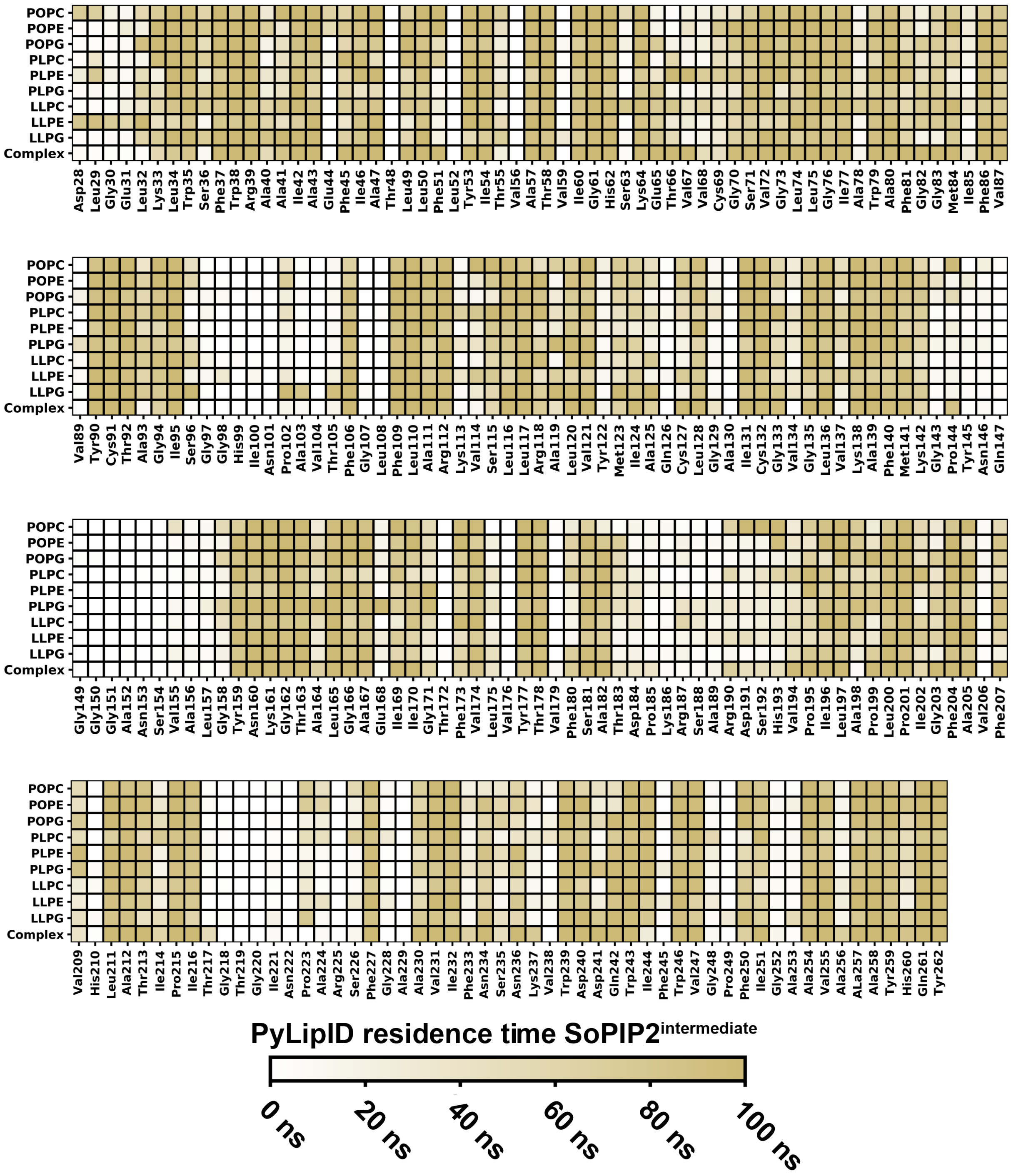


**Supplementary Figure 26. PyLipID average residence time for SoPIP2:lipid interactions along intermediate states.** The colorbar reports lipid residence time in nanoseconds based off calculations performed on continuous trajectories representing metastable states.

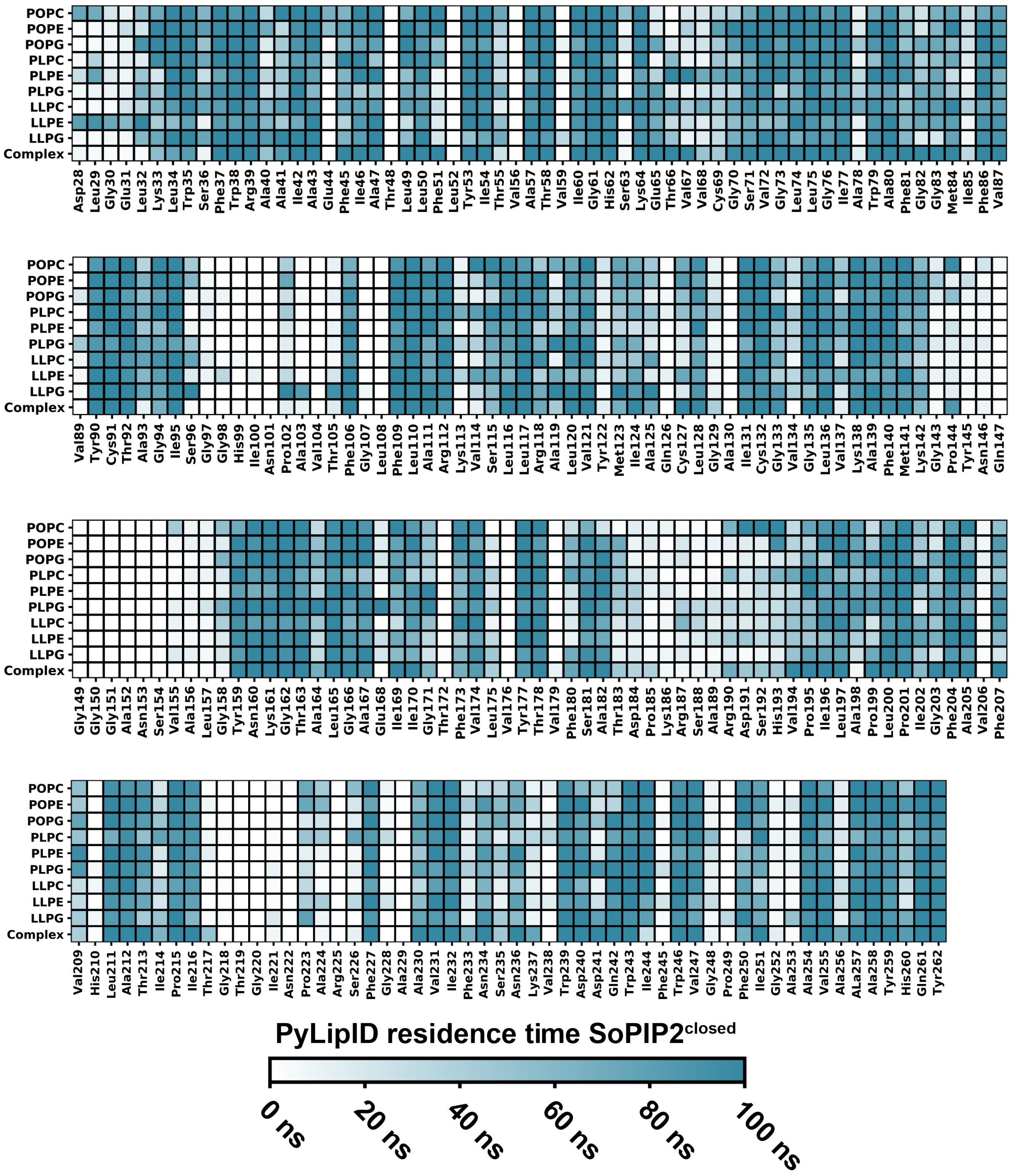


**Supplementary Figure 27. PyLipID average residence time for SoPIP2:lipid interactions along the closed state.** The colorbar reports lipid residence time in nanoseconds based off calculations performed on continuous trajectories representing metastable states.

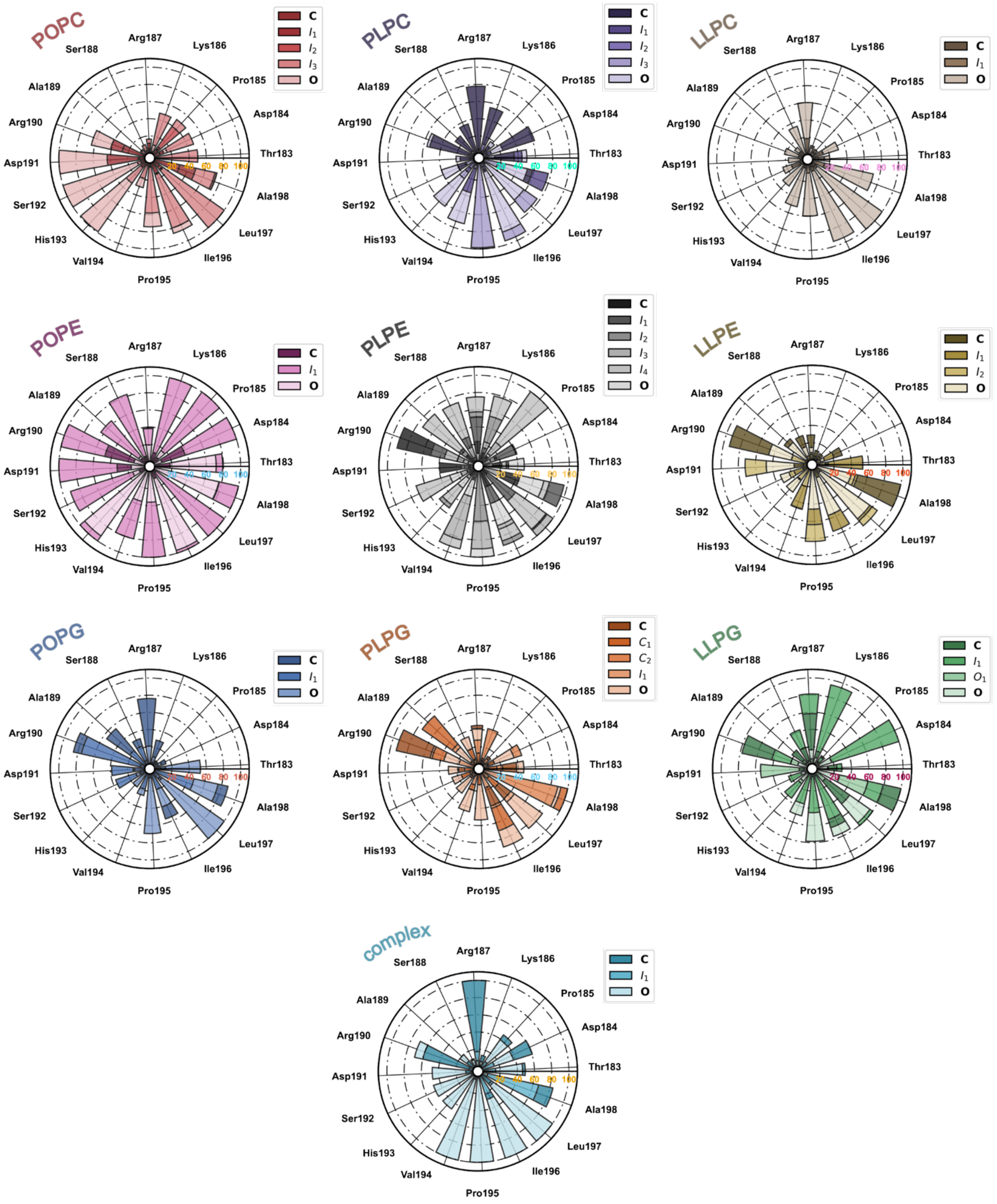


**Supplementary Figure 28. Radial fingerprints for loop D lipid binding interactions.** The angular axis depicts bar spokes representing each residue in loop D. The radial axis describes the PyLipID residence time for which lipids in each bilayer are bound to the selected residue, expressed in nanoseconds. Data representing each metastable state identity are colored accordingly.

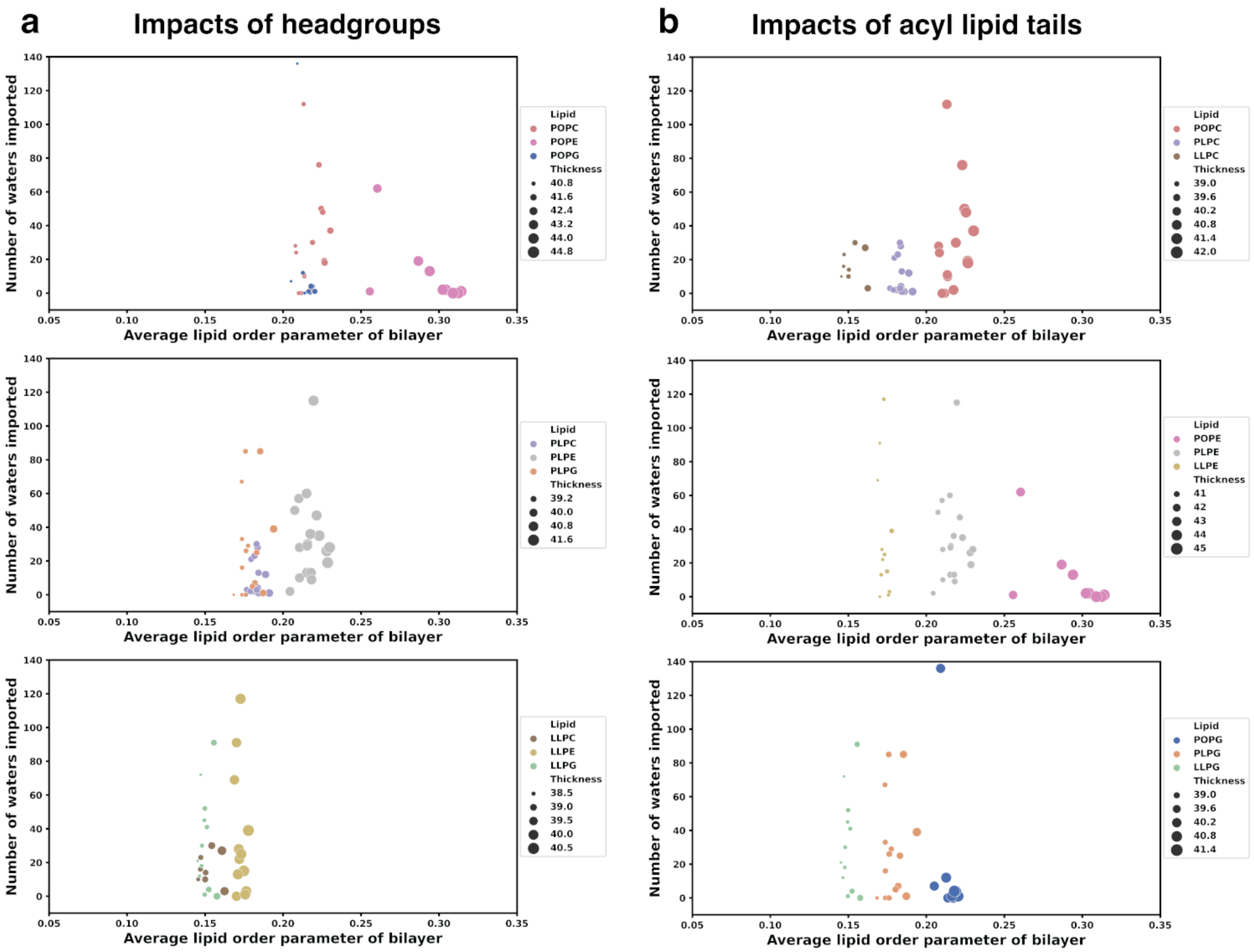


**Supplementary Figure 29. Dissecting influence of lipid headgroups (a) or acyl chains (b) on SoPIP2;1 protein function.** Colors represent each homogeneous lipid bilayer system. Dot sizes correspond to the average thickness of the membrane bilayer in each 100-ns trajectory.

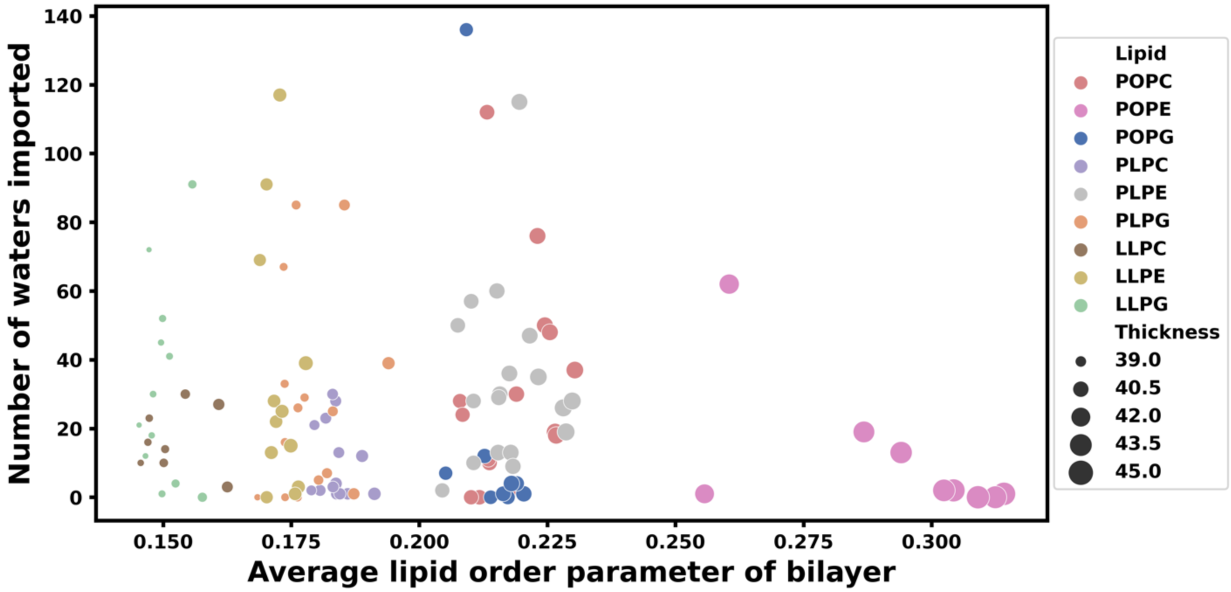


**Supplementary Figure 30. Number of waters imported versus average lipid order parameter for all selected trajectories belonging to each SoPIP2:bilayer macrostate.** Colors represent each homogeneous lipid bilayer system. Dot sizes correspond to the average thickness of the membrane bilayer in each 100-ns trajectory.

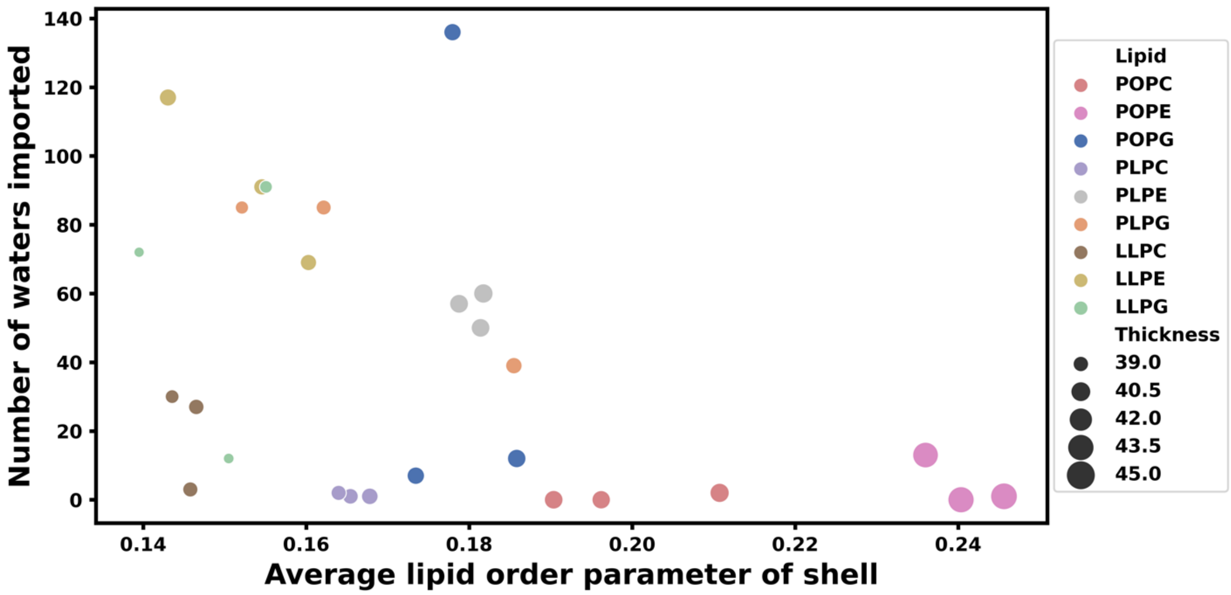


**Supplementary Figure 31. Number of waters imported versus the average lipid order parameter of the annular shell lipids of the open states.**

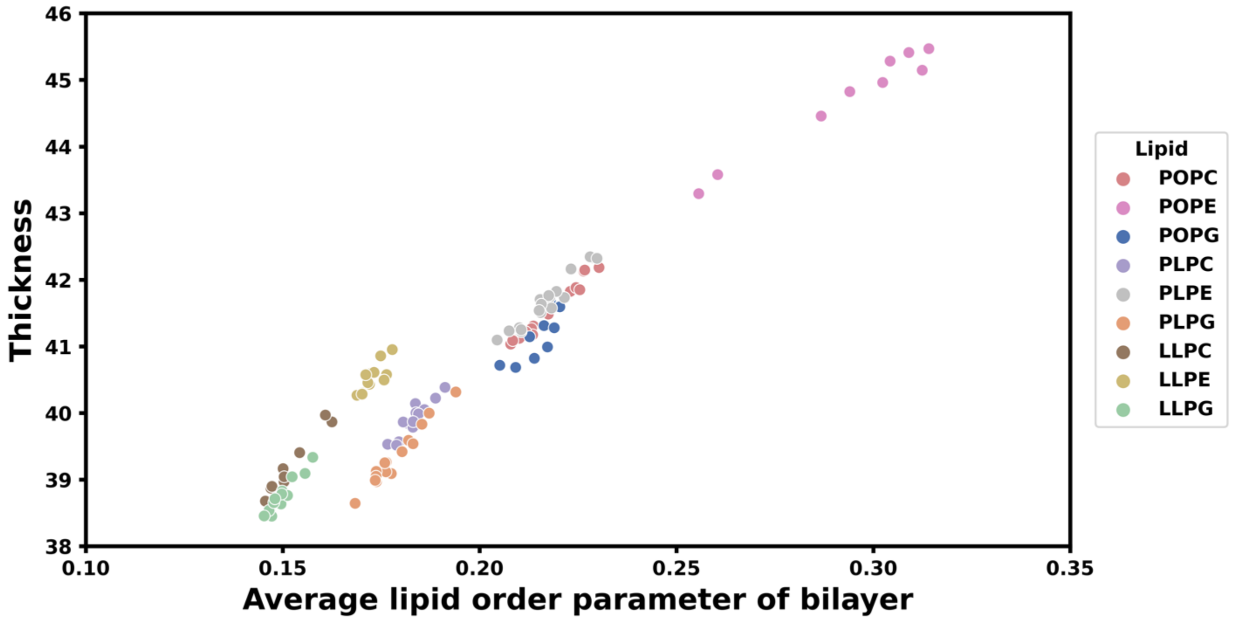


**Supplementary Figure 32. Correlation between the thickness and order parameter of the lipids for each homogeneous SoPIP2:bilayer macrostate.**

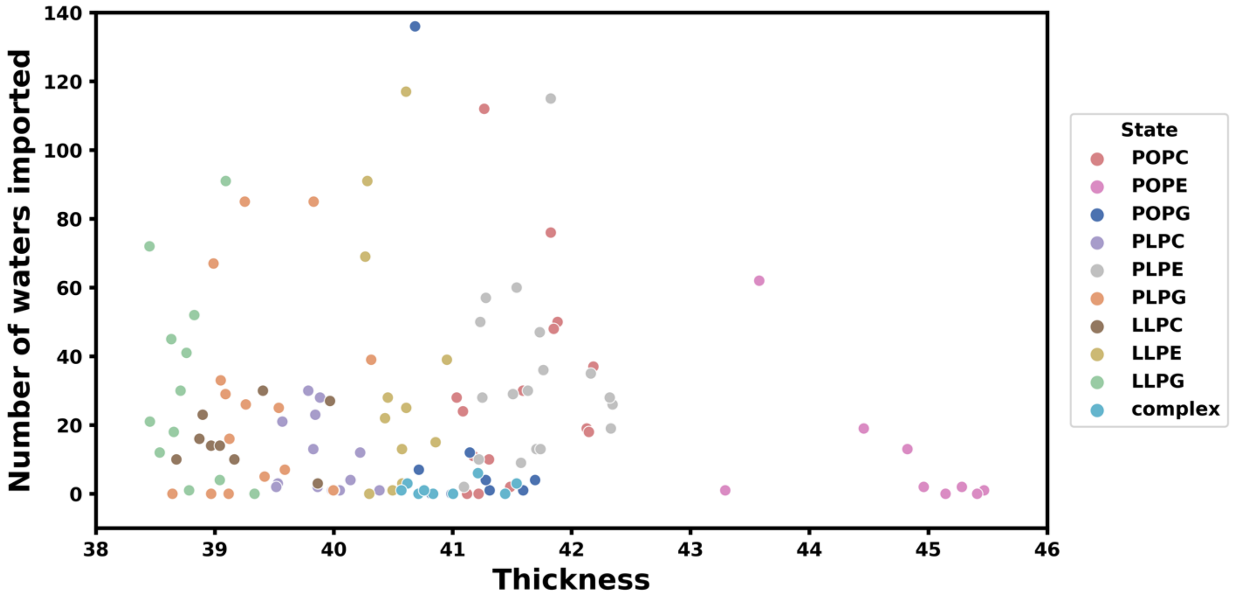


**Supplementary Figure 33. Number of waters imported versus thickness for each SoPIP2:bilayer macrostate.**

**Reporting Statistics for Main Text Figures**

<u>Main Text Figure 3 statistics</u>

*Open-to-Closed MFPT*

**POPC**:

Mean: 458.8834 $\mu$s
SEM: 27.9843 $\mu$s
Max: 2903.4981 $\mu$s
Min: 330.8550 $\mu$s
Q1: 408.5359 $\mu$s
Q3: 587.1626 $\mu$s
IQR: 178.6267 $\mu$s

*Closed-to-Open MFPT*

**POPC**:

Mean: 321.2135 $\mu$s
SEM: 152.8330 $\mu$s
Max: 22167.7741 $\mu$s
Min: 168.7478 $\mu$s
Q1: 284.5323 $\mu$s
Q3: 540.6944 $\mu$s
IQR: 256.1621 $\mu$s

*Open-to-Closed MFPT*

**POPE**:

Mean: 72.0186 $\mu$s
SEM: 4.9474 $\mu$s
Max: 749.0107 $\mu$s
Min: 42.7367 $\mu$s
Q1: 64.3303 $\mu$s
Q3: 108.9118 $\mu$s
IQR: 44.5815 $\mu$s

*Closed-to-Open MFPT*

**POPE**:

Mean: 101.5265 $\mu$s
SEM: 3.5662 $\mu$s
Max: 434.4697 $\mu$s
Min: 67.3931 $\mu$s
Q1: 101.1254 $\mu$s
Q3: 147.2240 $\mu$s
IQR: 46.0986 $\mu$s

*Open-to-Closed MFPT*

**POPG**:

Mean: 52.0677 $\mu$s
SEM: 2.6209 $\mu$s
Max: 464.2770 $\mu$s
Min: 33.5860 $\mu$s
Q1: 46.6788 $\mu$s
Q3: 66.0946 $\mu$s
IQR: 19.4159 $\mu$s

*Closed-to-Open MFPT*

**POPG**:

Mean: 37.5166 $\mu$s
SEM: 1.5825 $\mu$s
Max: 285.0786 $\mu$s
Min: 25.3451 $\mu$s
Q1: 34.4624 $\mu$s
Q3: 45.0999 $\mu$s
IQR: 10.6375 $\mu$s

*Open-to-Closed MFPT*

**PLPC**:

Mean: 33.6631 $\mu$s
SEM: 2.1656 $\mu$s
Max: 262.5888 $\mu$s
Min: 25.9399 $\mu$s
Q1: 30.5502 $\mu$s
Q3: 54.0463 $\mu$s
IQR: 23.4961 $\mu$s

*Closed-to-Open MFPT*

**PLPC**:

Mean: 19.3036 $\mu$s
SEM: 0.3756 $\mu$s
Max: 74.8096 $\mu$s
Min: 15.0060 $\mu$s
Q1: 17.9881 $\mu$s
Q3: 22.7372 $\mu$s
IQR: 4.7491 $\mu$s

Main Text Figure 3 statistics (cont.)

*Open-to-Closed MFPT*
**PLPE**:
Mean: 26.8867 $\mu$s
SEM: 0.6250 $\mu$s
Max: 75.3289 $\mu$s
Min: 17.8739 $\mu$s
Q1: 26.5421 $\mu$s
Q3: 37.2024 $\mu$s
IQR: 10.6603 $\mu$s

*Open-to-Closed MFPT*
**PLPG**:
Mean: 19.5279 $\mu$s
SEM: 2.0688 $\mu$s
Max: 167.6971 $\mu$s
Min: 14.6316 $\mu$s
Q1: 18.4096 $\mu$s
Q3: 25.8412 $\mu$s
IQR: 7.4316 $\mu$s

*Open-to-Closed MFPT*
**LLPC**:
Mean: 245.2923 $\mu$s
SEM: 23.5628 $\mu$s
Max: 2194.9861 $\mu$s
Min: 108.4483 $\mu$s
Q1: 186.5695 $\mu$s
Q3: 299.5613 $\mu$s
IQR: 112.9918 $\mu$s

*Open-to-Closed MFPT*
**LLPE**:
Mean: 51.4706 $\mu$s
SEM: 3.4186 $\mu$s
Max: 527.9734 $\mu$s
Min: 35.3516 $\mu$s
Q1: 45.2944 $\mu$s
Q3: 71.1791 $\mu$s
IQR: 25.8847 $\mu$s

*Closed-to-Open MFPT*
**PLPE**:
Mean: 16.1492 $\mu$s
SEM: 0.5214 $\mu$s
Max: 85.5535 $\mu$s
Min: 11.9728 $\mu$s
Q1: 15.5997 $\mu$s
Q3: 21.8781 $\mu$s
IQR: 6.2784 $\mu$s

*Closed-to-Open MFPT*
**PLPG**:
Mean: 35.7600 $\mu$s
SEM: 2.3007 $\mu$s
Max: 244.4715 $\mu$s
Min: 23.0654 $\mu$s
Q1: 32.3074 $\mu$s
Q3: 47.2155 $\mu$s
IQR: 14.9081 $\mu$s

*Closed-to-Open MFPT*
**LLPC**:
Mean: 231.7163 $\mu$s
SEM: 25.5303 $\mu$s
Max: 2796.6542 $\mu$s
Min: 122.8758 $\mu$s
Q1: 179.8494 $\mu$s
Q3: 274.1761 $\mu$s
IQR: 94.3267 $\mu$s

*Closed-to-Open MFPT*
**LLPE**:
Mean: 33.2353 $\mu$s
SEM: 0.5565 $\mu$s
Max: 82.9725 $\mu$s
Min: 23.7824 $\mu$s
Q1: 30.0367 $\mu$s
Q3: 39.2293 $\mu$s
IQR: 9.1926 $\mu$s

Main Text Figure 3 statistics (cont.)

*Open-to-Closed MFPT*
**LLPG**:
Mean: 13.4778 $\mu$s
SEM: 1.1938 $\mu$s
Max: 130.5756 $\mu$s
Min: 10.4394 $\mu$s
Q1: 13.1358 $\mu$s
Q3: 26.0189 $\mu$s
IQR: 12.8832 $\mu$s

*Closed-to-Open MFPT*
**LLPG**:
Mean: 49.9291 $\mu$s
SEM: 63.4698 $\mu$s
Max: 7921.9302 $\mu$s
Min: 36.8919 $\mu$s
Q1: 47.7134 $\mu$s
Q3: 72.9377 $\mu$s
IQR: 25.2243 $\mu$s

*Open-to-Closed MFPT*
**complex**:
Mean: 25.1357 $\mu$s
SEM: 0.3621 $\mu$s
Max: 48.1121 $\mu$s
Min: 19.4503 $\mu$s
Q1: 23.1758 $\mu$s
Q3: 28.7648 $\mu$s
IQR: 5.5889 $\mu$s

*Closed-to-Open MFPT*
**complex**:
Mean: 35.0892 $\mu$s
SEM: 0.9690 $\mu$s
Max: 104.4012 $\mu$s
Min: 22.6444 $\mu$s
Q1: 30.8460 $\mu$s
Q3: 41.7455 $\mu$s
IQR: 10.8996 $\mu$s

Main Text Figure 4c statistics

**POPC dihedral angles**

*Closed*:
Mean: -0.3791 π
SEM: 0.0017 π
Max: -0.2094 π
Min: -0.6385 π
Q1: -0.4087 π
Q3: -0.3473 π
IQR: 0.0614 π

*Intermediate-1*:
Mean: -0.3597 π
SEM: 0.0015 π
Max: -0.1948 π
Min: -0.5133 π
Q1: -0.3899 π
Q3: -0.3286 π
IQR: 0.0613 π

*Intermediate-2*:
Mean: -0.3365 π
SEM: 0.0017 π
Max: -0.1253 π
Min: -0.4767 π
Q1: -0.3721 π
Q3: -0.3074 π
IQR: 0.0647 π

*Intermediate-3*:
Mean: 0.5055 π
SEM: 0.0064 π
Max: 0.9913 π
Min: -0.9985 π
Q1: 0.4400 π
Q3: 0.5715 π
IQR: 0.1314 π

*Open*:
Mean: -0.3152 π
SEM: 0.0244 π
Max: 0.9986 π
Min: -0.9999 π
Q1: -0.8717 π
Q3: 0.6479 π
IQR: 1.5197 π

Main Text Figure 4c statistics (cont.)

**POPE dihedral angles**

| *Closed*: | *Intermediate-1*: | *Open*: |
|---|---|---|
| Mean: -0.3396 π | Mean: 0.5933 π | Mean: 0.6896 π |
| SEM: 0.0017 π | SEM: 0.0029 π | SEM: 0.0111 π |
| Max: -0.1477 π | Max: 0.9713 π | Max: 0.9994 π |
| Min: -0.5472 π | Min: 0.4103 π | Min: -0.9938 π |
| Q1: -0.3751 π | Q1: 0.5273 π | Q1: 0.6822 π |
| Q3: -0.3073 π | Q3: 0.6427 π | Q3: 0.8250 π |
| IQR: 0.0678 π | IQR: 0.1154 π | IQR: 0.1428 π |

**POPG dihedral angles**

| *Closed*: | *Intermediate-1*: | *Open*: |
|---|---|---|
| Mean: -0.3372 π | Mean: -0.2902 π | Mean: 0.4796 π |
| SEM: 0.0017 π | SEM: 0.0015 π | SEM: 0.0021 π |
| Max: -0.1798 π | Max: -0.1582 π | Max: 0.8311 π |
| Min: -0.5241 π | Min: -0.4882 π | Min: 0.2990 π |
| Q1: -0.3746 π | Q1: -0.3184 π | Q1: 0.4306 π |
| Q3: -0.2989 π | Q3: -0.2557 π | Q3: 0.5198 π |
| IQR: 0.0758 π | IQR: 0.0627 π | IQR: 0.0892 π |

**PLPC dihedral angles**

| *Closed*: | *Intermediate-1*: | *Intermediate-2*: |
|---|---|---|
| Mean: -0.3289 π | Mean: -0.3351 π | Mean: -0.3462 π |
| SEM: 0.0017 π | SEM: 0.0016 π | SEM: 0.0016 π |
| Max: -0.1624 π | Max: -0.1617 π | Max: -0.1845 π |
| Min: -0.4868 π | Min: -0.4686 π | Min: -0.6188 π |
| Q1: -0.3653 π | Q1: -0.3718 π | Q1: -0.3833 π |
| Q3: -0.2913 π | Q3: -0.2974 π | Q3: -0.3115 π |
| IQR: 0.0739 π | IQR: 0.0744 π | IQR: 0.0718 π |

| *Intermediate-3*: | *Open*: |
|---|---|
| Mean: 0.4511 π | Mean: 0.3920 π |
| SEM: 0.0038 π | SEM: 0.0034 π |
| Max: 0.8995 π | Max: 0.9083 π |
| Min: -0.9476 π | Min: 0.1255 π |
| Q1: 0.3891 π | Q1: 0.3307 π |
| Q3: 0.5052 π | Q3: 0.4282 π |
| IQR: 0.1162 π | IQR: 0.0975 π |

Main Text Figure 4c statistics (cont.)

**PLPE dihedral angles**

*Closed*:
Mean: -0.3469 π
SEM: 0.0016 π
Max: -0.1766 π
Min: -0.4846 π
Q1: -0.3838 π
Q3: -0.3124 π
IQR: 0.0714 π

*Intermediate-1*:
Mean: -0.2965 π
SEM: 0.0023 π
Max: -0.0647 π
Min: -0.4914 π
Q1: -0.3495 π
Q3: -0.2509 π
IQR: 0.0986 π

*Intermediate-2*:
Mean: 0.3927 π
SEM: 0.0085 π
Max: 0.8135 π
Min: -0.4673 π
Q1: 0.4156 π
Q3: 0.5294 π
IQR: 0.1138 π

*Intermediate-3*:
Mean: 0.5426 π
SEM: 0.0034 π
Max: 0.8891 π
Min: -0.9883 π
Q1: 0.4991 π
Q3: 0.5852 π
IQR: 0.0861 π

*Intermediate-4*:
Mean: 0.6322 π
SEM: 0.0034 π
Max: 0.9489 π
Min: 0.3097 π
Q1: 0.5464 π
Q3: 0.7122 π
IQR: 0.1659 π

*Open*:
Mean: 0.5011 π
SEM: 0.0094 π
Max: 0.9989 π
Min: -0.9989 π
Q1: 0.4033 π
Q3: 0.6765 π
IQR: 0.2732 π

**PLPG dihedral angles**

*Closed*:
Mean: -0.1867 π
SEM: 0.0084 π
Max: 0.4185 π
Min: -0.5351 π
Q1: -0.3657 π
Q3: 0.1616 π
IQR: 0.5273 π

*Closed-1*:
Mean: -0.3471 π
SEM: 0.0016 π
Max: -0.1654 π
Min: -0.5025 π
Q1: -0.3776 π
Q3: -0.3184 π
IQR: 0.05910 π

*Closed-2*:
Mean: -0.3619 π
SEM: 0.0016 π
Max: -0.1880 π
Min: -0.5689 π
Q1: -0.3964 π
Q3: -0.3329 π
IQR: 0.0635 π

*Intermediate-1*:
Mean: 0.2497 π
SEM: 0.0079 π
Max: 0.7237 π
Min: -0.4824 π
Q1: 0.1761 π
Q3: 0.4318 π
IQR: 0.2557 π

*Open*:
Mean: 0.4927 π
SEM: 0.0078 π
Max: 0.9945 π
Min: -0.9980 π
Q1: 0.4138 π
Q3: 0.6037 π
IQR: 0.1900 π

Main Text Figure 4c statistics (cont.)

**LLPC dihedral angles**

*Closed*:
- Mean: -0.3367 π
- SEM: 0.0016 π
- Max: -0.1463 π
- Min: -0.5068 π
- Q1: -0.3697 π
- Q3: -0.3076 π
- IQR: 0.0621 π

*Intermediate-1*:
- Mean: -0.0070 π
- SEM: 0.0034 π
- Max: 0.4474 π
- Min: -0.3012 π
- Q1: -0.0641 π
- Q3: 0.0213 π
- IQR: 0.0855 π

*Open*:
- Mean: 0.5600 π
- SEM: 0.0031 π
- Max: 0.9072 π
- Min: 0.3271 π
- Q1: 0.4843 π
- Q3: 0.6224 π
- IQR: 0.1381 π

**LLPE dihedral angles**

*Closed*:
- Mean: -0.3592 π
- SEM: 0.0019 π
- Max: -0.1884 π
- Min: -0.5780 π
- Q1: -0.4000 π
- Q3: -0.3195 π
- IQR: 0.0805 π

*Intermediate-1*:
- Mean: 0.5775 π
- SEM: 0.0054 π
- Max: 0.9720 π
- Min: -0.9871 π
- Q1: 0.4887 π
- Q3: 0.6652 π
- IQR: 0.1765 π

*Intermediate-2*:
- Mean: 0.5228 π
- SEM: 0.0024 π
- Max: 0.9006 π
- Min: 0.1936 π
- Q1: 0.4740 π
- Q3: 0.5664 π
- IQR: 0.0924 π

*Open*:
- Mean: 0.2298 π
- SEM: 0.0155 π
- Max: 0.9510 π
- Min: -0.5308 π
- Q1: -0.3492 π
- Q3: 0.6351 π
- IQR: 0.9843 π

Main Text Figure 4c statistics (cont.)

**LLPG dihedral angles**

| *Closed*: | *Intermediate-1*: | *Intermediate-2*: |
| --- | --- | --- |
| Mean: -0.3628 π | Mean: 0.2155 π | Mean: 0.0818 π |
| SEM: 0.0017 π | SEM: 0.0092 π | SEM: 0.0033 π |
| Max: -0.2254 π | Max: 0.7016 π | Max: 0.3964 π |
| Min: -0.5235 π | Min: -0.4591 π | Min: -0.1936 π |
| Q1: -0.4002 π | Q1: 0.1479 π | Q1: -0.0016 π |
| Q3: -0.3235 π | Q3: 0.4362 π | Q3: 0.1593 π |
| IQR: 0.0768 π | IQR: 0.2883 π | IQR: 0.1609 π |

*Open*:

Mean: 0.5614 π
SEM: 0.0062 π
Max: 0.9764 π
Min: -0.9994 π
Q1: 0.5082 π
Q3: 0.6398 π
IQR: 0.1316 π

**Complex dihedral angles**

| *Closed*: | *Intermediate-1*: | *Open*: |
| --- | --- | --- |
| Mean: -0.3673 π | Mean: -0.3798 π | Mean: 0.5005 π |
| SEM: 0.0018 π | SEM: 0.0016 π | SEM: 0.0025 π |
| Max: -0.2034 π | Max: -0.2154 π | Max: 0.8573 π |
| Min: -0.5267 π | Min: -0.6024 π | Min: 0.1063 π |
| Q1: -0.4067 π | Q1: -0.4111 π | Q1: 0.4547 π |
| Q3: -0.3302 π | Q3: -0.3485 π | Q3: 0.5476 π |
| IQR: 0.0765 π | IQR: 0.0626 π | IQR: 0.0929 π |

Main Text Figure 5a

**POPC number of waters transported**

| *Open-like states (number waters*: | *Closed-like states (number waters)*: |
| --- | --- |
| Mean: 10.5000 | Mean: 44.6670 |
| SEM: 4.4850 | SEM: 10.8280 |
| Max: 24.0000 | Max: 112.0000 |
| Min: 0.0000 | Min: 10.0000 |
| Q1: 0.5000 | Q1: 28.0000 |
| Q3: 18.7500 | Q3: 50.0000 |
| IQR: 18.2500 | IQR: 22.0000 |

Main Text Figure 5a (cont.)

**POPE number of waters transported**

| *Open-like states (number waters*: | *Closed-like states (number waters)*: |
|---|---|
| Mean: 3.0000 | Mean: 27.3330 |
| SEM: 2.0330 | SEM: 18.0950 |
| Max: 13.0000 | Max: 62.0000 |
| Min: 0.0000 | Min: 1.0000 |
| Q1: 0.2500 | Q1: 10.0000 |
| Q3: 2.0000 | Q3: 40.5000 |
| IQR: 1.7500 | IQR: 30.5000 |

**POPG number of waters transported**

| *Open-like states (number waters*: | *Closed-like states (number waters)*: |
|---|---|
| Mean: 27.3330 | Mean: 0.3330 |
| SEM: 21.7860 | SEM: 0.3330 |
| Max: 136.0000 | Max: 1.0000 |
| Min: 1.0000 | Min: 0.0000 |
| Q1: 4.0000 | Q1: 0.0000 |
| Q3: 10.7500 | Q3: 0.5000 |
| IQR: 6.7500 | IQR: 0.5000 |

**PLPC number of waters transported**

| *Open-like states (number waters*: | *Closed-like states (number waters)*: |
|---|---|
| Mean: 2.8890 | Mean: 19.8330 |
| SEM: 1.1720 | SEM: 3.9950 |
| Max: 12.0000 | Max: 30.0000 |
| Min: 1.0000 | Min: 4.0000 |
| Q1: 1.0000 | Q1: 15.0000 |
| Q3: 3.0000 | Q3: 26.7500 |
| IQR: 2.0000 | IQR: 11.7500 |

**PLPE number of waters transported**

| *Open-like states (number waters*: | *Closed-like states (number waters)*: |
|---|---|
| Mean: 42.7500 | Mean: 15.6670 |
| SEM: 7.5430 | SEM: 6.4790 |
| Max: 115.0000 | Max: 47.0000 |
| Min: 19.0000 | Min: 2.0000 |
| Q1: 28.0000 | Q1: 9.2500 |
| Q3: 51.7500 | Q3: 13.0000 |
| IQR: 23.7500 | IQR: 3.7500 |

<u>Main Text Figure 5a (cont.)</u>

**PLPG number of waters transported**

*Open-like states (number waters*:
Mean: 55.6670
SEM: 10.9260
Max: 85.0000
Min: 25.0000
Q1: 34.5000
Q3: 80.5000
IQR: 46.0000

*Closed-like states (number waters)*:
Mean: 9.3330
SEM: 3.8440
Max: 29.0000
Min: 0.0000
Q1: 0.0000
Q3: 16.0000
IQR: 16.0000

**LLPC number of waters transported**

*Open-like states (number waters*:
Mean: 16.3330
SEM: 4.2010
Max: 30.0000
Min: 3.0000
Q1: 11.0000
Q3: 23.7500
IQR: 12.7500

*Closed-like states (number waters)*:
Mean: 16.3330
SEM: 3.7560
Max: 23.0000
Min: 10.0000
Q1: 13.0000
Q3: 19.5000
IQR: 6.5000

**LLPE number of waters transported**

*Open-like states (number waters*:
Mean: 46.5560
SEM: 12.3780
Max: 117.0000
Min: 13.0000
Q1: 22.0000
Q3: 69.0000
IQR: 47.0000

*Closed-like states (number waters)*:
Mean: 1.3330
SEM: 0.8820
Max: 3.0000
Min: 0.0000
Q1: 0.5000
Q3: 2.0000
IQR: 1.5000

**LLPG number of waters transported**

*Open-like states (number waters*:
Mean: 27.6670
SEM: 10.8030
Max: 91.0000
Min: 0.0000
Q1: 4.0000
Q3: 30.0000
IQR: 26.0000

*Closed-like states (number waters)*:
Mean: 46.0000
SEM: 3.2150
Max: 52.0000
Min: 41.0000
Q1: 43.0000
Q3: 48.5000
IQR: 5.5000

Main Text Figure 5a (cont.)

**Complex number of waters transported**

*Open-like states (number waters*:

Mean: 1.000
SEM: 1.000
Max: 3.000
Min: 0.0000
Q1: 0.0000
Q3: 1.5000
IQR: 1.5000

*Closed-like states (number waters)*:

Mean: 1.8330
SEM: 0.9460
Max: 6.0000
Min: 0.0000
Q1: 0.2500
Q3: 2.5000
IQR: 2.2500

Main Text Figure 7b

**POPC hydrophobic mismatch**

*Shell-bulk (open)*:

Mean: -3.8287 Å
SEM: 0.0560 Å
Max: 2.5476 Å
Min: -9.0610 Å
Q1: -5.0329 Å
Q3: -2.6520 Å
IQR: 2.3808 Å

*Protein-bulk (open)*:

Mean: -2.0222 Å
SEM: 0.0638 Å
Max: 3.1569 Å
Min: -11.1682 Å
Q1: -3.2790 Å
Q3: -0.6991 Å
IQR: 2.5799 Å

*Shell-bulk (closed)*:

Mean: -4.6434 Å
SEM: 0.0639 Å
Max: 2.6689 Å
Min: -9.9257 Å
Q1: -5.9986 Å
Q3: -3.3941 Å
IQR: 2.6045 Å

*Protein-bulk (closed)*:

Mean: -4.4084 Å
SEM: 0.0585 Å
Max: 1.1002 Å
Min: -9.7127 Å
Q1: -5.6514 Å
Q3: -3.1145 Å
IQR: 2.5369 Å

Main Text Figure 7b (cont.)

**POPE hydrophobic mismatch**

*Shell-bulk (open)*:

Mean: -4.8398 Å
SEM: 0.0586 Å
Max: 0.9876 Å
Min: -10.3360 Å
Q1: -6.2181 Å
Q3: -3.5692 Å
IQR: 2.6489 Å

*Protein-bulk (open)*:

Mean: -8.2255 Å
SEM: 0.0629 Å
Max: -1.4700 Å
Min: -14.1722 Å
Q1: -9.5563 Å
Q3: -6.9365 Å
IQR: 2.6198 Å

*Shell-bulk (closed)*:

Mean: -5.0148 Å
SEM: 0.0717 Å
Max: 3.5053 Å
Min: -12.6789 Å
Q1: -6.5339 Å
Q3: -3.4458 Å
IQR: 3.0880 Å

*Protein-bulk (closed)*:

Mean: -7.6059 Å
SEM: 0.0610 Å
Max: -1.5461 Å
Min: -12.7630 Å
Q1: -8.9080 Å
Q3: -6.2473 Å
IQR: 2.6607 Å

**POPG hydrophobic mismatch**

*Shell-bulk (open)*:

Mean: -3.7844 Å
SEM: 0.0602 Å
Max: 5.0473 Å
Min: -9.5781 Å
Q1: -5.0843 Å
Q3: -2.7489 Å
IQR: 2.3354 Å

*Protein-bulk (open)*:

Mean: -3.3904 Å
SEM: 0.0546 Å
Max: 1.3500 Å
Min: -8.9380 Å
Q1: -4.3961 Å
Q3: -2.2212 Å
IQR: 2.1749 Å

*Shell-bulk (closed)*:

Mean: -3.6377 Å
SEM: 0.0717 Å
Max: 3.9751 Å
Min: -10.2105 Å
Q1: -5.0256 Å
Q3: -2.2557 Å
IQR: 2.7699 Å

*Protein-bulk (closed)*:

Mean: -4.2882 Å
SEM: 0.0669 Å
Max: 3.4152 Å
Min: -10.8284 Å
Q1: -5.7791 Å
Q3: -2.8882 Å
IQR: 2.8909 Å

Main Text Figure 7b (cont.)

**PLPC hydrophobic mismatch**

*Shell-bulk (open)*:
Mean: -1.4877 Å
SEM: 0.0624 Å
Max: 7.8821 Å
Min: -6.8068 Å
Q1: -2.7635 Å
Q3: -0.2628 Å
IQR: 2.5007 Å

*Protein-bulk (open)*:
Mean: 0.4571 Å
SEM: 0.0582 Å
Max: 5.6552 Å
Min: -5.9491 Å
Q1: -0.7738 Å
Q3: 1.7753 Å
IQR: 2.5491 Å

*Shell-bulk (closed)*:
Mean: -1.9596 Å
SEM: 0.0704 Å
Max: 4.8924 Å
Min: -8.9098 Å
Q1: -3.4795 Å
Q3: -0.4824 Å
IQR: 2.9971 Å

*Protein-bulk (closed)*:
Mean: -2.3030 Å
SEM: 0.0585 Å
Max: 3.4705 Å
Min: -8.5723 Å
Q1: -3.5831 Å
Q3: -1.0685 Å
IQR: 2.5146 Å

**PLPE hydrophobic mismatch**

*Shell-bulk (open)*:
Mean: -2.1980 Å
SEM: 0.0658 Å
Max: 5.5800 Å
Min: -8.9110 Å
Q1: -3.5885 Å
Q3: -0.8916 Å
IQR: 2.6969 Å

*Protein-bulk (open)*:
Mean: -6.5753 Å
SEM: 0.0646 Å
Max: 0.0848 Å
Min: -11.2414 Å
Q1: -8.0887 Å
Q3: -5.2673 Å
IQR: 2.8214 Å

*Shell-bulk (closed)*:
Mean: -2.5067 Å
SEM: 0.0643 Å
Max: 6.0858 Å
Min: -7.4761 Å
Q1: -3.9251 Å
Q3: -1.2195 Å
IQR: 2.7055 Å

*Protein-bulk (closed)*:
Mean: -5.4049 Å
SEM: 0.0707 Å
Max: 0.6428 Å
Min: -12.4528 Å
Q1: -6.9770 Å
Q3: -3.7895 Å
IQR: 3.1876 Å

Main Text Figure 7b (cont.)

**PLPG hydrophobic mismatch**

*Shell-bulk (open)*:
- Mean: -2.2977 Å
- SEM: 0.0637 Å
- Max: 3.6597 Å
- Min: -8.4652 Å
- Q1: -3.6694 Å
- Q3: -1.0275 Å
- IQR: 2.6419 Å

*Protein-bulk (open)*:
- Mean: -3.0195 Å
- SEM: 0.0751 Å
- Max: 4.3473 Å
- Min: -10.4995 Å
- Q1: -4.7033 Å
- Q3: -1.3459 Å
- IQR: 3.3574 Å

*Shell-bulk (closed)*:
- Mean: -2.8988 Å
- SEM: 0.0673 Å
- Max: 5.7877 Å
- Min: -9.8809 Å
- Q1: -4.1710 Å
- Q3: -1.5283 Å
- IQR: 2.6427 Å

*Protein-bulk (closed)*:
- Mean: -2.9424 Å
- SEM: 0.0631 Å
- Max: 2.4119 Å
- Min: -9.1227 Å
- Q1: -4.3366 Å
- Q3: -1.5291 Å
- IQR: 2.8075 Å

**LLPC hydrophobic mismatch**

*Shell-bulk (open)*:
- Mean: -1.6597 Å
- SEM: 0.0668 Å
- Max: 6.2484 Å
- Min: -7.4418 Å
- Q1: -3.1732 Å
- Q3: -0.2942 Å
- IQR: -1.7888 Å

*Protein-bulk (open)*:
- Mean: -3.6772 Å
- SEM: 0.0646 Å
- Max: 2.9606 Å
- Min: -9.6430 Å
- Q1: -5.0936 Å
- Q3: -2.2287 Å
- IQR: 2.8649 Å

*Shell-bulk (closed)*:
- Mean: -0.9719 Å
- SEM: 0.0745 Å
- Max: 7.2639 Å
- Min: -7.9944 Å
- Q1: -2.5308 Å
- Q3: 0.4421 Å
- IQR: 2.9729 Å

*Protein-bulk (closed)*:
- Mean: -1.0293 Å
- SEM: 0.0647 Å
- Max: 4.5630 Å
- Min: -7.1777 Å
- Q1: -2.4813 Å
- Q3: 0.4017 Å
- IQR: 2.8830 Å

Main Text Figure 7b (cont.)

**LLPE hydrophobic mismatch**

*Shell-bulk (open)*:
- Mean: -2.3811 Å
- SEM: 0.0670 Å
- Max: 5.2149 Å
- Min: -8.4881 Å
- Q1: -3.8677 Å
- Q3: -0.9387 Å
- IQR: 2.9290 Å

*Protein-bulk (open)*:
- Mean: -3.8156 Å
- SEM: 0.0689 Å
- Max: 2.7755 Å
- Min: -11.2984 Å
- Q1: -5.2817 Å
- Q3: -2.2906 Å
- IQR: 2.9911 Å

*Shell-bulk (closed)*:
- Mean: -2.2506 Å
- SEM: 0.0683 Å
- Max: 4.6229 Å
- Min: -8.5588 Å
- Q1: -3.7800 Å
- Q3: -0.8978 Å
- IQR: 2.8821 Å

*Protein-bulk (closed)*:
- Mean: -3.2102 Å
- SEM: 0.0539 Å
- Max: 3.9319 Å
- Min: -8.6914 Å
- Q1: -4.2768 Å
- Q3: -2.1791 Å
- IQR: 2.0977 Å

**LLPG hydrophobic mismatch**

*Shell-bulk (open)*:
- Mean: -2.3736 Å
- SEM: 0.0638 Å
- Max: 4.3777 Å
- Min: -8.8440 Å
- Q1: -3.7453 Å
- Q3: -1.0455 Å
- IQR: 2.6998 Å

*Protein-bulk (open)*:
- Mean: -1.1372 Å
- SEM: 0.0553 Å
- Max: 4.3586 Å
- Min: -6.3533 Å
- Q1: -2.2352 Å
- Q3: -0.0810 Å
- IQR: 2.1542 Å

*Shell-bulk (closed)*:
- Mean: -1.5512 Å
- SEM: 0.0654 Å
- Max: 5.5395 Å
- Min: -6.9454 Å
- Q1: -3.0256 Å
- Q3: -0.1642 Å
- IQR: 2.8614 Å

*Protein-bulk (closed)*:
- Mean: -2.4528 Å
- SEM: 0.0493 Å
- Max: 2.0333 Å
- Min: -6.9780 Å
- Q1: -3.6268 Å
- Q3: -1.3256 Å
- IQR: 2.3012 Å

Main Text Figure 7b (cont.)

**Complex hydrophobic mismatch**

*Shell-bulk (open)*:

Mean: -0.5737 Å
SEM: 0.0577 Å
Max: 6.1358 Å
Min: -6.6802 Å
Q1: -1.7435 Å
Q3: 0.5640 Å
IQR: 2.3076 Å

*Shell-bulk (closed)*:

Mean: -3.3613 Å
SEM: 0.0554 Å
Max: 2.9191 Å
Min: -8.2139 Å
Q1: -4.5989 Å
Q3: -2.1971 Å
IQR: 2.4017 Å

*Protein-bulk (open)*:

Mean: -7.7258 Å
SEM: 0.0490 Å
Max: -2.4300 Å
Min: -12.1461 Å
Q1: -8.8418 Å
Q3: -6.6848 Å
IQR: 2.1570 Å

*Protein-bulk (closed)*:

Mean: -5.2528 Å
SEM: 0.0495 Å
Max: -0.0367 Å
Min: -8.6757 Å
Q1: -6.4006 Å
Q3: -4.1443 Å
IQR: 2.2563 Å
